# A Review on Breathing Behaviors of Metal-Organic-Frameworks (MOFs) for Gas Adsorption

**DOI:** 10.3390/ma7043198

**Published:** 2014-04-21

**Authors:** Mays Alhamami, Huu Doan, Chil-Hung Cheng

**Affiliations:** Department of Chemical Engineering, Ryerson University, 350 Victoria Street, Toronto, ON M5B 2K3, Canada, E-Mails: mays.alhamami@ryerson.ca (M.A.); hdoan@ryerson.ca (H.D.)

**Keywords:** metal-organic frameworks (MOFs), breathing, gas adsorption, gas storage, flexibility, post-synthetic modifications (PSM)

## Abstract

Metal-organic frameworks (MOFs) are a new class of microporous materials that possess framework flexibility, large surface areas, “tailor-made” framework functionalities, and tunable pore sizes. These features empower MOFs superior performances and broader application spectra than those of zeolites and phosphine-based molecular sieves. In parallel with designing new structures and new chemistry of MOFs, the observation of unique breathing behaviors upon adsorption of gases or solvents stimulates their potential applications as host materials in gas storage for renewable energy. This has attracted intense research energy to understand the causes at the atomic level, using *in situ* X-ray diffraction, calorimetry, Fourier transform infrared spectroscopy, and molecular dynamics simulations. This article is developed in the following order: first to introduce the definition of MOFs and the observation of their framework flexibility. Second, synthesis routes of MOFs are summarized with the emphasis on the hydrothermal synthesis, owing to the environmental-benign and economically availability of water. Third, MOFs exhibiting breathing behaviors are summarized, followed by rationales from thermodynamic viewpoint. Subsequently, effects of various functionalities on breathing behaviors are appraised, including using post-synthetic modification routes. Finally, possible framework spatial requirements of MOFs for yielding breathing behaviors are highlighted as the design strategies for new syntheses.

## 1. Introduction

Metal-organic frameworks (MOFs) are a new class of crystalline microporous materials that exhibit tunable functionalities, large surface areas, framework flexibility, catalytic activities [1], and gas separation capability among others [2]. Their frameworks are formed by the covalent linkages between metals or metal oxides and organic moieties. Even though, compared with zeolites, MOFs suffer a major drawback of relatively lower thermal stability, the flexible structure of MOFs contrast to rigid zeolite frameworks, yielding the unique breathing phenomena or gate-opening effect. The pore diameter of MOFs enlarges or shrinks while external stimuli are applied or removed, such as gas molecules, solvents, or pressure [3]. The features of breathing phenomena and large surface areas enable MOFs suitable materials for applications in green and renewable energy as media of gas storages, sensing and separation [4–6]. Scientists and engineers strive to understand and substantiate the origin of the framework flexibility such that properties of MOFs can be designed at the synthesis stage. Given that a large variety of MOFs family and publications, this review focuses on summarizing the MOFs’ breathing effect, to which a hydrothermal synthesis route is applied. Readers can find more information in a critical review published recently by Férey’s group, in which the authors elucidated rationales of the breathing effects [7]. This review article will describe the breathing phenomena of MOFs in following sequences: (1) definition of MOFs and summary of synthesis conditions; (2) summary of MOFs exhibiting the breathing phenomena; (3) post-synthetic medication to alter breathing phenomena; (4) outlooks of MOFs’ breathing phenomena.

## 2. What Are MOFs?

**Figure 1.**
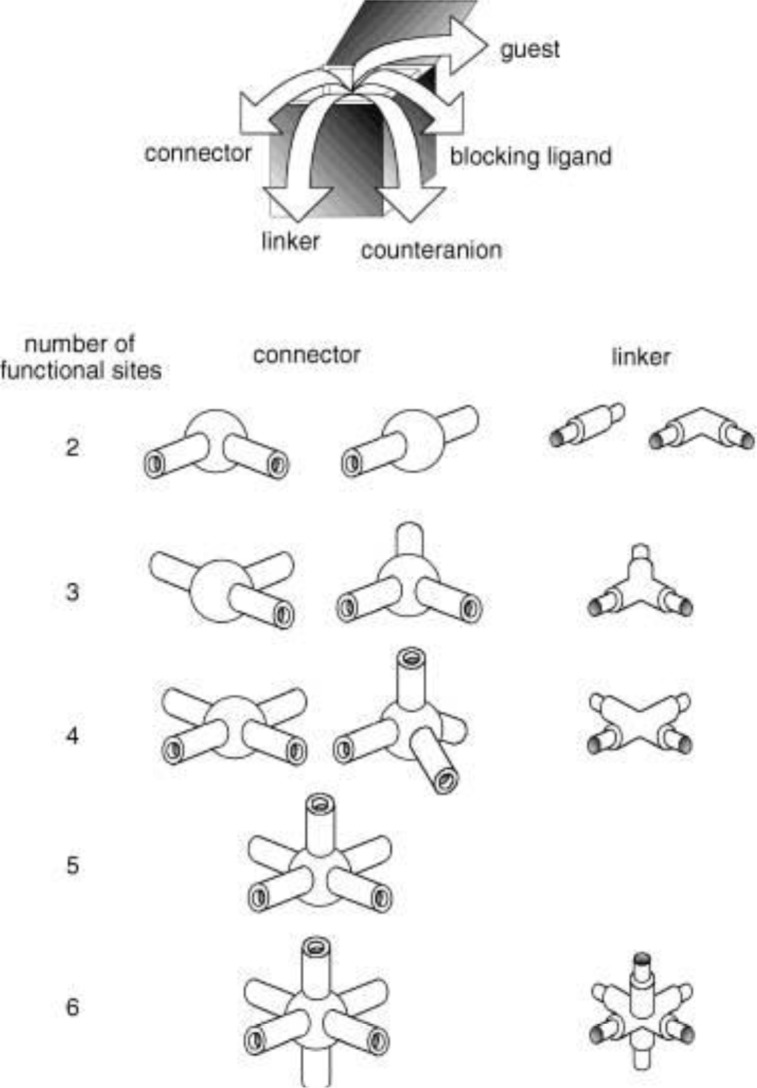
Geometrics of metal ions and organic ligands. Reprinted with permission from [9]. Copyright 2004 WILEY-VCH Verlag GmbH & Co. KGaA, Weinheim.

**Figure 2.**
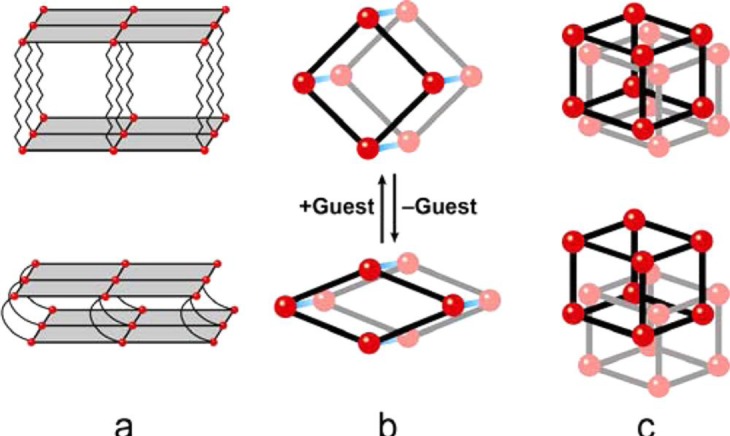
The illustration dynamic behaviors of the metal-organic frameworks’ (MOFs) structure upon interacting with guest molecules. (**a**) 2-dimension; (**b**) 1-dimension; (**c**) 3 dimension. Red spheres stand for the metals; lines stand for the organic linkers. Reprinted with permission from [11]. Copyright 2013 American Chemical Society.

Metal-organic frameworks (MOFs), or called metal-ligand coordination polymers, are organic-inorganic hybrid extended networks that are constructed via covalent linkages between metal ions/metal clusters and organic ligands. The strategies of forming versatile frameworks utilize the principles of reticular synthesis [8]. The majority of metal ions/metal clusters are transition metals with various geometries, due to their versatile coordination numbers. These geometries include square–planer, tetrahedron, and octahedron among others [9]. The organic ligands contain halides, cyanides, neutral organic molecules (4,4′-bipyridine), and anionic organic molecules (benzenedicarboxylic acid). Each constituent is schematically drawn in Figure 1. Together, both organic and inorganic components can form *n*-dimensional (*n* = 1, 2, and 3) motifs which show flexibility upon interactions with guest molecules, schematically shown in Figure 2 [7,10–12].

Kitagawa’s group classifies three types of MOFs exhibiting flexibility [10]. For the first type, rigid 2D layers are covalently connected by flexible pillars. Upon the absorption of guest molecules, the elongation of pillars leads to the expansion of frameworks. Vice versa, frameworks shrink upon the removal of guest molecules (Figure 2a). Regarding the second type, the framework topology remains the same upon the adsorption of guest molecules. The rotation of organic moieties, caused by strong guest-host interactions, induces the volume change (Figure 2b). In the case of the third type, the interpenetrated 3D grids slide apart, causing the pores open or close upon the adsorption of guest molecules (Figure 2c).

The first coordination polymer, Ni(CN)_2_(NH_3_)·C_6_H_6_, was synthesized by Hofmann and Küspert in 1897 [13], called Hofmann Complex. The coordination polymer was formed by reacting slowly of C_6_H_6_ with Ni(CN)_2_ in a NH_3_ solution. The structure of coordination polymer was later refined using X-ray crystallography, as a two-dimensional layer structure formed by covalent linkages between Ni and CN groups [14]. The parallel layers are held up by extruding NH_3_ groups, forming interplanar cavities in which reside benzene molecules. The work inspired research towards designing various inorganic-organic hybrid polymers using diamines, such as 4,4′-bipyridine. The adoption of longer chain length of organic linkers in MOFs’ framework enlarged the interplanar space by 33%, from 8.28 Å to 10.9 Å. The framework with longer organic ligands creates larger cavities for molecule encapsulation.

**Figure 3.**
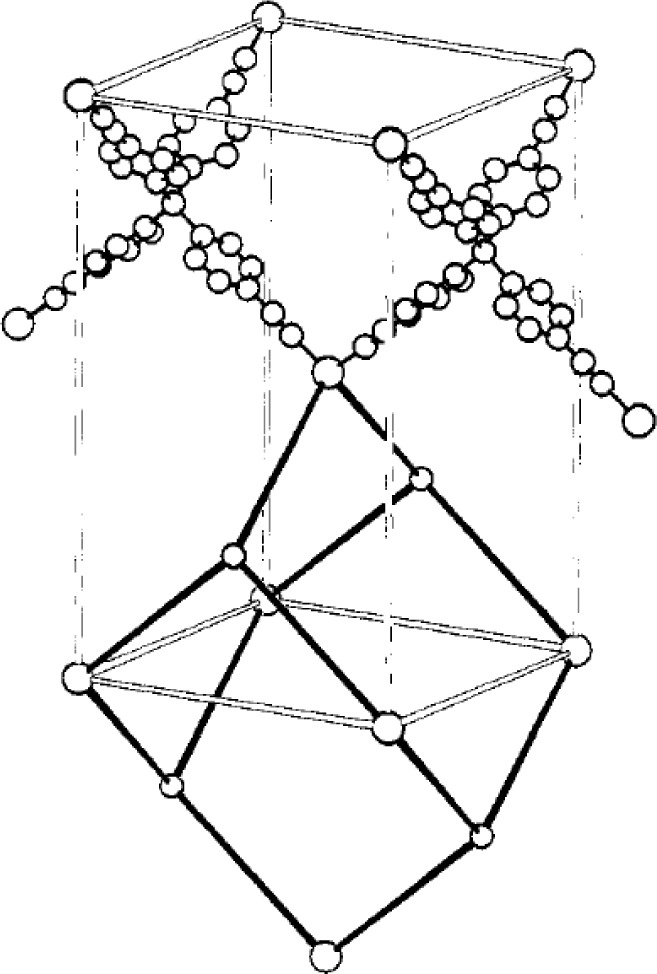
Crystallographic structure of coordination polymer, Cu^I^[C(C_6_H_4_·CN)_4_]_n_. An adamantine-like cavity is highlighted using bold lines. Reprinted with permission from [15]. Copyright 1989 American Chemical Society.

In addition to aforementioned bidentate linear organic molecules, other multidentate organic linkers, such as 4,4′,4′′,4′′′-tetracyanotetraphenylmethane was first applied by Robson’s group to yield 3-dimensional infinite framework with a large unit cell volume of 4200 Å [15,16], in which about two-thirds of the volume is occupied by removable solvents molecules. The refined crystallographic structure is shown in Figure 3. Robson’s work shed the light of controlling the cavity size of MOFs with predetermined size and shape of inorganic nodes, and organic linkers.

Researches on MOFs have seen explosively increasing since 2000 [9,17]. In the open literature, there are several families of MOFs systematically synthesized and coined their nomenclatures using the abbreviation of institution’s name. Pioneer works done by Yaghi and O’Keefe has named their resulting solids as MOF–*n* (where *n* = 1, 2, 3, and *etc.*) [8]. Others include MILs (Matériaux de l’lnstitut Lavoisier), HKUSTs (Hong-Kong University of Science and Technology), POST (Pohang University of Science and Technology), *etc*.

### 2.1. Single-Crystal-to-Single-Crystal *(*SCSC*)* Phenomena

The application of MOFs on gas adsorption was first reported using a three-dimensional framework: M_2_(4,4′-bpy)_3_(NO_3_)_4_·xH_2_O with M = Co, Ni, Zn and 4,4′-bpy = 4,4′-bipyridine [18]. The MOFs were synthesized at room temperature for 7 days in an acetone/EtOH mixture. Detail X-ray crystallography analyses indicated that the Co center forms pentagonal bipyramid geometry, bridged by 4,4′-bpy organic linkers. The linkage forms infinite 1-dimensional chains with an interdigited, or tongue-and-groove, structure. The cavities have a window size of 3 Å × 6 Å and 3 Å × 3 Å along *a* and *b* axes respectively, which were suitable for gas adsorption. The authors proposed that the adsorption feature evolved from the affinity of organic linkers inside the framework cavities. The adsorption properties can be further fine-tuned via the guest-host interaction using versatile organic linkers [19,20].

Kitagawa’s group adopted various framework regulators, AF_6_^−^ anions (A = Si, Ge, and P), to manipulate selectively the framework connectivity and pore dimensions upon hydrolysis. The resulted compounds demonstrated a higher adsorption capacity towards methane. It is proposed that the guest-host interaction and the size exclusion effect between the pore size and kinetic diameters of adsorbates lead to the selectivity of gas adsorption. However, it is noted that no structure transformation of MOFs was observed, meaning that the framework exhibits the rigidity upon the adsorption of gas molecules.

The guest-host interaction also accounts for the single-crystal-to-single-crystal (SCSC) transformation that was first successfully discovered in [Ni_2_(4,4′-bpy)_3_(NO_3_)_4_]·6EtOH upon the removal of solvent [21]. Their detail single crystal X-ray diffraction data indicated that the unit cell volume shrunk 2.3% after encapsulated ethanol was evaporated or removed from the channels while the framework crystallinity still remains integrated. At room temperature, the adsorptions of H_2_O, MeOH, and EtOH of the MOF were demonstrated to be reversible, showing the inhaling-exhaling cycle, despite the relative small difference of the unit cell volume. A similar behavior was noticed later using mixed organic ligands, 2,5-dicarboxypyridine (H_2_pydc) and 4,4′-bipyridine (4,4′-bpy). A MOF of [Fe(pydc)(4,4′-bpy)]·H_2_O was synthesized hydrothermally and was characterized as a non-interpenetrated square-grid structure with a cavity size of 11.5 Å × 8.8 Å [22]. The authors reported a larger magnitude of volume contraction upon the removal of solvent, 6.6%–8.2%, possibly owing to strong edge-to-face aromatic π–π stacking interactions between the aromatic groups of pydc and 4,4′-bpy in adjacent layers [22]. The doubly-bridged layer structure exhibits its robustness and sliding of layers during the SCSC transformation.

**Figure 4.**
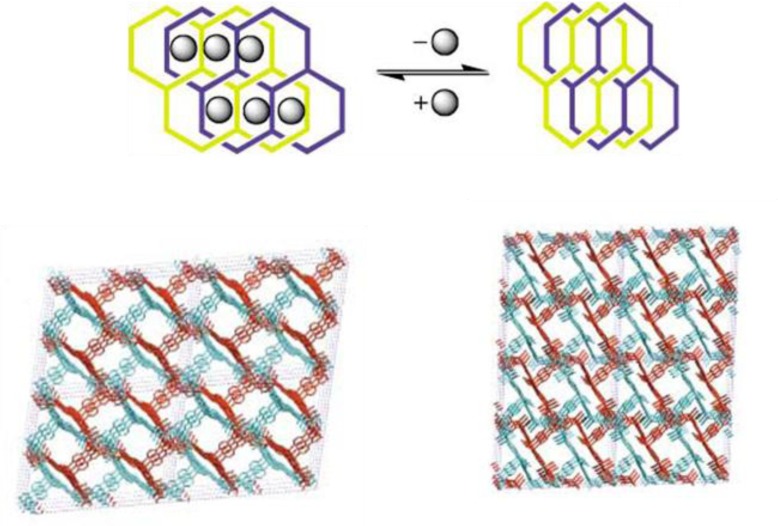
The crystal structure of {(ZnI_2_)_3_(TPT)_2_·6C_6_H_5_NO_2_}_n_. (Left) the net formed by ten molecules of TPT and ten Zn atoms. (Right) the interpenetrated framework viewed along the *b*-axis. C: gray, N: blue, Zn: magenta. Reprinted with permission from [23]. Copyright© 2002 WILEY-VCH Verlag GmbH & Co. KGaA, Weinheim.

**Figure 5.**
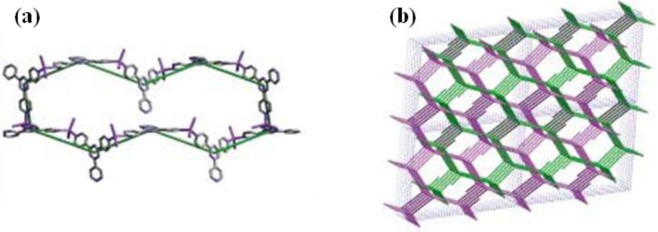
Schematic drawing of SCSC of {(ZnI_2_)_3_(TPT)_2_·6C_6_H_5_NO_2_}_n_ upon the removal of nitrobenzene as the guest molecules. The contraction of unit cell volumes is viewed along the (100) direction. Reprinted with permission from [23]. Copyright© 2002 WILEY-VCH Verlag GmbH & Co. KGaA, Weinheim.

The unit cell volume change of MOF crystals caused by the SCSC transformation becomes noticeable in {(ZnI_2_)_3_(TPT)_2_·6C_6_H_5_NO_2_}_n_, where TPT stands for 2,4,6-tris(4-pyridyl)triazine. The geometry of zinc atoms is tetrahedral, linking to two iodine atoms and two TPT molecules individually. The network adopts a (10,3)-b configuration, in which contains 10 TPT molecules and 10 zinc atoms, forming an interpenetrated 3-D framework, shown in Figure 4. The randomly-packed nitrobenzene molecules occupy 60% of the unit cell volume. The Bravais lattice of the interpenetrated framework changes from *monoclinic* to *triclinic* upon the removal of solvent, yielding the unit cell volume contraction of 20%–23% [23]. The schematic diagram is illustrated in Figure 5. The shrinkage of unit cell volume is owing to shorter distances between Zn-Zn and ligand-ligand, changing from 6.9 Å to 5.2 Å and 8.2 Å to 5.1 Å respectively. It is noteworthy that the MOF exhibited a *reversible* swelling behavior as solvent re-entered the channels and showed a wide range of chemical stability upon exposing to various organic solvents, such as mesitylene, and *cis*-stilbene among others. This type of flexibility evolved from the crystal structure transformation is classified as the breathing behavior [24].

**Figure 6.**
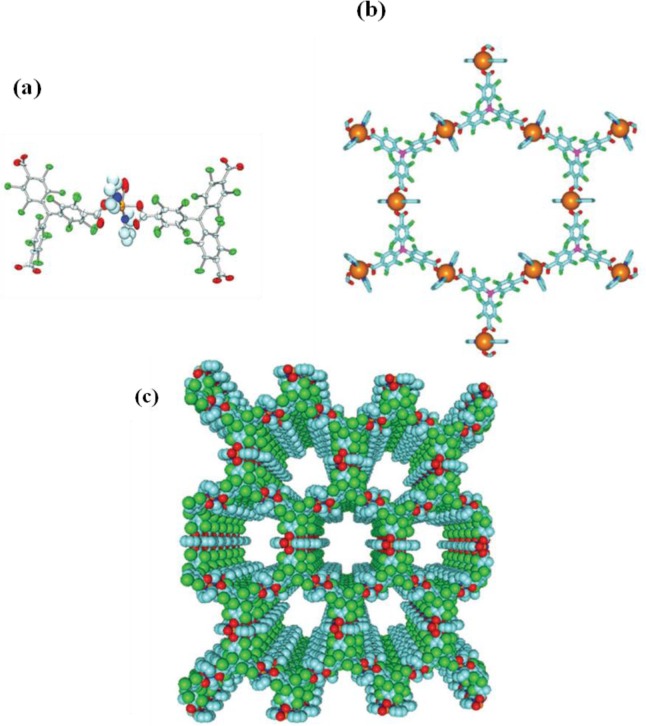
(**a**) The structure of copper(II) tricarboxylate; (**b**) the connection of building blocks to form a hexagonal pore structure; (**c**) the mesoporous structure formed by the stacking of hexagonal pore structure along (001) direction. Cu atom: orange; C atom: light blue and violet; O atom: red; Cl atom: green; and N atom: navy. Reprinted with permission from [25], Copyright 2003 Macmillan Publishers Ltd: Nature Materials.

A similar solvent-induced reversible framework transition was reported for MOROF-1, a mesoporous MOF of which a polychlorinated triphenylmethyl radicals(PTM), functionalized with three carboxylic groups (TC), was used as the organic ligand (PTMTC), illustrated in Figure 6a [25]. The adoption of the multidentate bulky linkers was designated for the formation of open framework with even larger pore diameter. The copper metal centers adopt a geometry of square pyramidal polyhedrons, and link with ligands, forming a two-dimensional honeycomb layer structure (Figure 6b). Different layers are organized via π–π and van der Waals interactions, yielding an open framework (Figure 6c). The window sizes measured along (001) and (100) directions are about 2.8–3.1 nm and 0.5–0.7 nm, respectively. The MOF experienced a huge volume contraction, 25%–35%, upon the removal of solvent and became an amorphous closed-pore material. The MOF exhibited high selectivity towards methanol and ethanol than other several organic solvents upon re-adsorption.

The occurrence of SCSC transformation upon the solvent removal and re-adsorption clearly indicates the flexibility of metal-organic frameworks. The feature is unique and has not been observed in rigid porous materials, such as zeolites. Some authors called this phenomena “breathing”, which is described as a reversible structural transition of organic-inorganic hybrid materials whose unit cell dimensions experience “large variations (>5 Å)” upon the exposure of external stimuli [26,27]. Given their framework flexibility and selectivity of solvent re-adsorption, it is generally recognized that the flexible MOFs would find larger applications in gas separation, sequestration and purification if they exhibit similar breathing behaviors while gas molecules are external stimuli.

### 2.2. Hydrothermal Synthesis versus Solvothermal Synthesis

Syntheses of Metal-organic frameworks can take place at room temperature, or using solvothermal synthesis, microwave-assisted synthesis, electrochemical synthesis, or sonochemical synthesis among others [28,29]. Being the most prevail approach to form novel solid state materials by materials scientists and chemists, the solvothermal synthesis can be carried out using H_2_O or organic solvents as the medium [30]. The majority of this article focuses on the formations of MOFs from aqueous solutions, in contrast to those from organic solvents, because the former case is easy to scale-up for mass production, cost-effective, and environmental benign in terms of solvent [31,32].

Solvothermal techniques are widely applied to synthesize novel metal oxides materials from a reaction mixture inside a closed reaction vessel, under the supercritical temperature of the solvent which can be either aqueous or organic liquids [33,34]. Some researchers reckon the processes as hydrothermal syntheses while H_2_O is used as the solvent [35,36]. The main purposes of solvothermal techniques are to widen the synthesis avenue for synthesizing new materials as well as to manipulate the shape of material crystals. Compared with other synthesis methods such as ceramic, sol-gel techniques, the main advantages of solvothermal techniques are (1) simple one-pot/one-step preparation/synthesis; (2) ability to control the morphology of material crystals; (3) efficient synthesis conditions, with milder synthesis temperature and shorter synthesis duration, possibly due to reduced viscosity and dielectric constants [37].

Syntheses of MOFs originally adopted the precipitation route with careful diffusion control or slow evaporation of organic ligands and solvent molecules [15]. Later, syntheses of MOFs take up the solvothermal synthesis pathway which can overcome the solubility and integrity concerns of a desired secondary building unit (SBU) generated *in situ* in the reaction mixture. However, the structure of yielded materials is sensitive to subtle changes in concentration, solvent polarity, pH and temperature.

**Figure 7.**
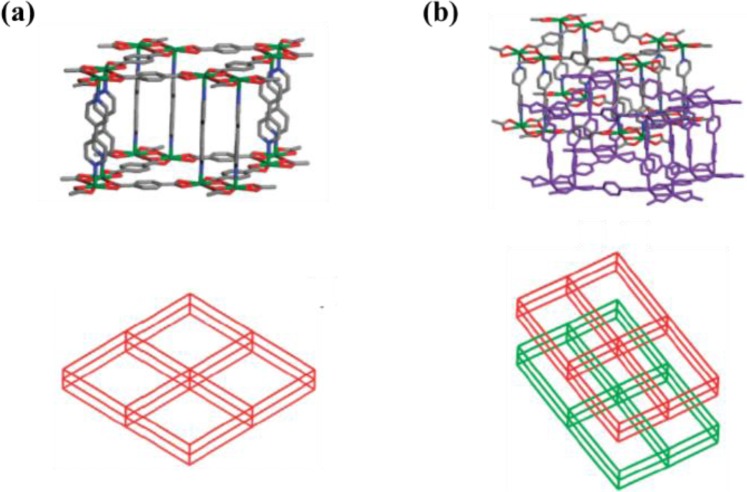
(**a**) Non-interpenetrated structure of [Cd(bipy)(bdc)] formed using low concentration of reactants; (**b**) Interpenetrated structure of [Cd(bipy)(bdc)] formed using high concentration of reactants. Reprinted with permission from [39]. Copyright 2009 American Chemical Society.

The concentration of starting materials determined the structure of final products. For MOF-5 system, the dilution of reactants leads to the formation of non-interpenetrated structure (IRMOF-10, -12, -14, and -16), subsequently possessing higher porosity compared with individual concentrated counterpart [38]. A similar concentration-dependent trend of MOF structure was observed in the primitive cubic type (*pcu*) MOF of [Cd(bipy)(bdc)]·3DMF·H_2_O, whereas bipy = 4,4′-bipyridine, and bdc = 1,4-benzenedicarboxylic acid, shown in Figure 7 [39]. It is speculated that the formation of a sub-lattice in the voids of non-interpenetrated structures can be further reduced while lowering the reactant concentrations.

The pH of starting precursor solutions plays significant roles at the structure of final products as well. From the system of Al^3+^/H_4_btec/H_2_O/NaOH, where H_4_btec represents pyromellitic acid, MIL-118 and MIL-121 were formed under low pH environment, while MIL-120 was the preferable phase at higher pH synthesis conditions [40]. It was proposed, based on detail crystallography analyses, that corner-sharing [AlO_6_] octahedra were formed under low pH solutions; however, edge-sharing alumina polyhedra were observed at high pH environment.

**Figure 8.**
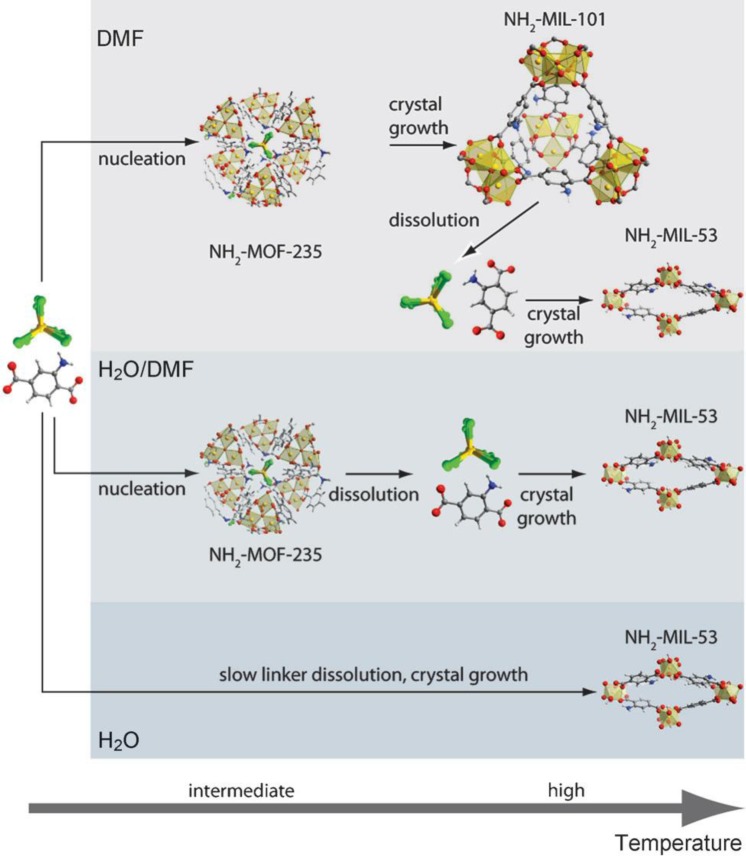
Schematic diagram of the effect of solvent polarity on final structure of MOF. C: gray; H: white; N: blue; O: red; Al: yellow; Cl: green. Reprinted with permission from [44]. Copyright© 2011 WILEY-VCH Verlag GmbH & Co. KGaA, Weinheim.

The polarity of solvent affects profoundly the final structure formation, perhaps via influencing the solubility of organic linkers as well as its proteolysis properties [41]. For the system of Fe^3+^/H_2_BDC-NH_2_/solvent (H_2_BDC-NH_2_ = 2-amino-terephthalic acid), only Fe-MIL-88B-NH_2_ was formed when acetonitrile and methanol were used as solvents [42]. Only Fe-MIL-53-NH_2_ was obtained if water was the solvent. However, a mixture Fe-MIL-88B-NH_2_ and Fe-MIL-101-NH_2_ was isolated when *N*,*N*’-dimethylformamide (DMF) was used as the solvent. Similarly, pure Cr-MIL-100 was obtained from Cr^3+^/H_3_BTC/H_2_O system (H_3_BTC = 1,3,5-benzenetricarboxylic acid), and Cr-MIL-96 co-existed with Cr-MIL-100 when a small quantity of methanol was added into H_2_O [43]. Further increase of methanol content led to the formation of pure Cr-MIL-96 phase. Regarding the Al^3+^/H_2_BDC-NH_2_/DMF system, a mixture of Al-MIL-53-NH_2_ and Al-MIL-101-NH_2_ was observed when DMF was used as the solvent. However, increasing the H_2_O content or using H_2_O as the sole solvent ensured the formation of pure Al-MIL-53-NH_2_ [44]. Using *in situ* X-ray scattering technique illustrated that the formation of an intermediate phase, MOF-235, stabilized by DMF, leads to the crystallization of Al-MIL-101-NH_2._ The presence of H_2_O causes the hydrolysis of MOF-235 phase, which subsequently facilitate the formation of Al-MIL-53-NH_2._ The schematic diagram of propose mechanism is illustrated in Figure 8.

As scientists and engineers envision the promising performance of MOFs at the gas separation and purification, syntheses of MOFs at a large scale arises as another challenge and attracts research attention [2,45,46]. It is widely recognized that several synthesis concerns have to be circumvented prior to practical applications, such as synthesis temperature and duration, yield, availability and cost of starting materials, synthesis steps, and quantity of solvents [28]. The role of solvent becomes critical and it is highly desired to minimize or avoid the use of organic solvents. Given this concern, this work mainly focuses on reviewing the breathing behaviors of MOFs synthesized via the hydrothermal route.

It is noteworthy that major MOFs are synthesized using organic solvents, except some MIL series. As summarized in Table 1, it is clearly demonstrated that the applied synthesis temperature is the highest among MOFs, and the synthesis duration is usually longer than its counterparts synthesized from organic solvents, which are summarized in Table 2.

## 3. Characterizations of Breathing Behaviors

**Figure 9.**
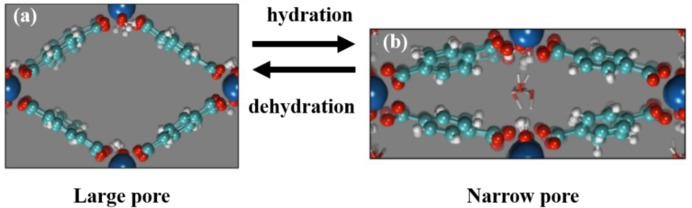
Illustration of the breathing behavior of MIL-53 using heat as the external stimulus. (**a**) dehydrated *lp* form; (**b**) hydrated *np* form. Reprinted with permission from [87]. Copyright 2013 American Chemical Society.

Despite the variety of MOFs, only several classes exhibit the unique breathing phenomena. For example, MIL-53 series are formed by connections of corner-sharing MO_4_(OH)_2_ octahedra linked by 1,4-benzenedicarboxylic (BDC) acids. Since MIL-53 (Al, Cr) are synthesized hydrothermally, denoted as *as*-*synthesized* MIL-53 (MIL-53*as*), the channels of MOFs are filled with disordered BDC and H_2_O molecules, demonstrating the narrow-pore (*np*) form. Shown in Figure 9, this *np* structure is caused by the hydrogen-bond interactions between the hydrogen atoms of the water molecules and the oxygen atoms of the carboxylic group and the μ_2_-hydroxo group [86]. Upon the dehydration at elevated temperatures, the MIL-53s exhibit a porous structure which is referred as the large-pore (*lp*) form, due to the absence of the interactions.

**Figure 10.**
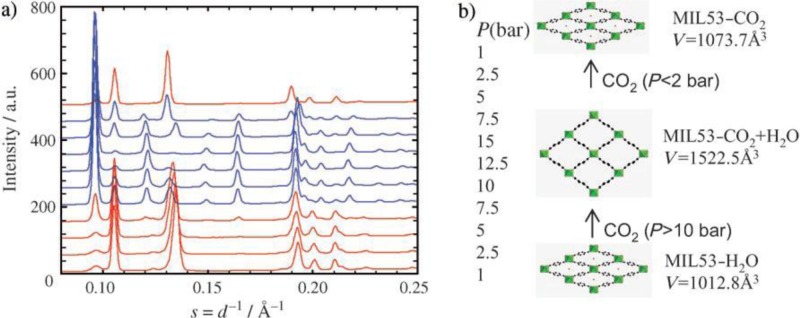
(**a**) The illustration of structural change of hydrated MIL-53(Cr) using *in situ* X-ray diffraction with respect to the pressure cycle of CO_2_; (**b**) Schematic diagram of corresponding breathing behavior of MIL-53(Cr). Reprinted with permission from [86]. Copyright 2006 WILEY-VCH Verlag GmbH & Co. KGaA, Weinheim.

An unprecedented adsorption behavior is observed as the hydrated MIL-53s are exposed to CO_2_ molecules. The hydrated MIL-53s remain the *np* form when CO_2_ pressure is below 5 bar. Further increasing the gas pressure turns the framework into the coexistence of *np* and *lp* forms. The framework turns into a pure *lp* form when the gas pressure reaches above 15 bar (about 7.2 mmol·g^−1^), shown in Figure 10. The unit cell volume expands from 1012.8 Å^3^ (hydrated) to 1522.5 Å^3^ (hydrated + CO_2_). This behavior is different from their dehydrated counterparts that exhibit two-step structure transition upon CO_2_ adsorption (*lp* → *np* → *lp*), during which the first plateau occurs at around 3 mmol·g^−1^ while the second plateau starts evolving around 8 bar (7.7 mmol·g^−1^). The lag of the structure transition might be due to the steric hindrance in the presence of H_2_O molecules prior to the CO_2_ adsorption. Furthermore, the hydrated MIL-53(Cr) shows the minute CH_4_ uptake (0.2 mmol·g^−1^ at 20 bar), compared to its dehydrated counterpart (4.6 mmol·g^−1^). This behavior is projected to have wide applications in gas storage and separation, such as the selective adsorption of CO_2_ over CH_4_ [88,89].

### 3.1. Porosity Measurements

The structural transformation of MOFs, displaying the breathing behavior, is induced by the dipole moment interaction between guest molecules and the host framework. The transition affects the gas adsorption capacity that can be quantified using porosity measurements. Measurements using powder X-ray diffraction (PXRD), and ^129^Xe NMR are conducted to unveil the structure transition at the atomic level, providing a comprehensive porosity information of the breathing dynamics [90].

**Figure 11.**
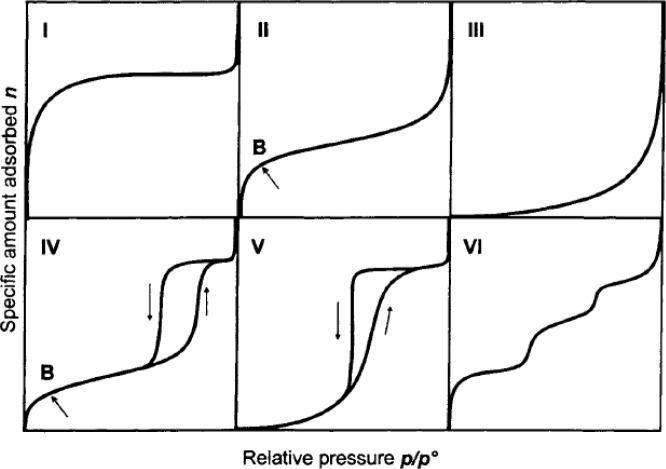
Six types of gas physisorption isotherms. Reprinted with permission from [100]. Copyright 1985 IUPAC.

The gas adsorption isotherms are classified by International Union of Pure and Applied Chemistry (IUPAC) into six types, shown in Figure 11 [91]. Type I is the Langmuir isotherm commonly observed in microporous materials, of which the steep increase of adsorbed quantity at low relative pressure indicates that the available microporous volume is occupied. Type-II, and -IV isotherms are possible similar materials, in which mesopores and macropores are present. In addition, the multi-layer adsorption might occur in the middle relative pressure range. The subtle difference between these two types of isotherms is the presence of hysteresis loop in Type IV, in which implies the occurrence of capillary condensation within mesopores, due to the strength of adsorbate–adsorbent and adsorbate–adsorbate interactions [24]. Type-III and -V isotherms indicate the weak adsorbate-adsorbent interactions. Type-VI isotherm might associate with layer-by-layer adsorption on a uniform surface.

**Figure 12.**
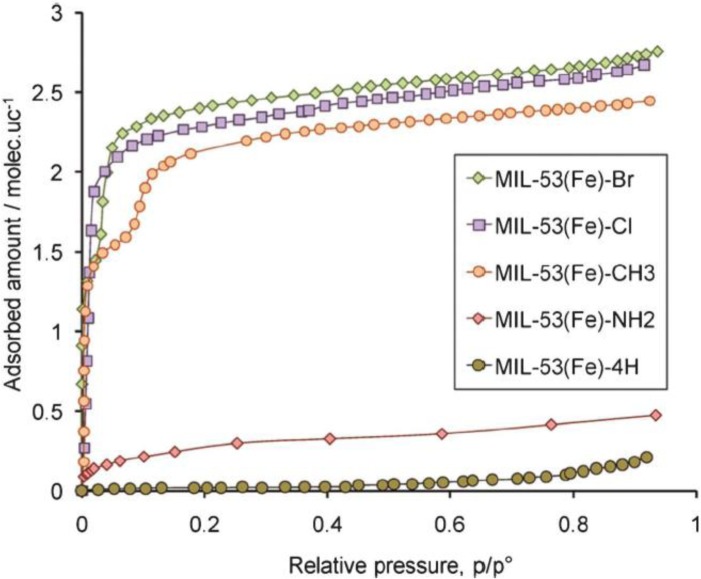
Adsorption isotherms of *n*-hexane of various modified MIL-53(Fe). MIL-53(Fe)-4H stands for the non-modified MIL-53(Fe). Reprinted with permission from [101]. Copyright 2011 American Chemical Society.

As aforementioned, dehydrated MIL-53 (Al, Cr) demonstrate the *lp* form. The N_2_ sorption measurements show Type I isotherms, indicating their microporosity [26,92,93]. However, dehydrated MIL-53 (Fe) and MIL-53 (Sc) display no microporosity towards the specific stimuli, as indicated in Figure 12 [94–96]. This is attributed to the presence of closed-pore (*cp*) form or very-narrow-pore (*vnp*) form of these two MIL-53s [97]. Similar Type I isotherm can be observed in the dehydrated MIL-47(V) [98], which is an isostructural analogue of MIL-53, formed by connecting V^4+^O_6_ octahedra with 1,4-benzenedicarboxylic (BDC) acids [99].

**Figure 13.**
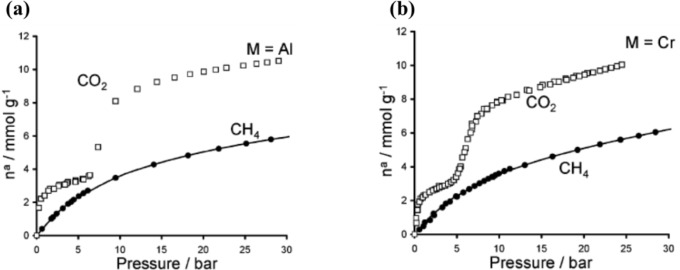
Isotherms at 304 K for the adsorption of CH_4_ and CO_2_. (**a**) MIL-53(Al); and (**b**) MIL-53(Cr). Reprinted with permission from [99]. Copyright 2005 American Chemical Society.

However, if CO_2_ is used as the adsorbate, a Type VI isotherm is noticed in Figure 13, in which a step or an inflection point can be observed. The presence of the inflection point in isotherms could be evolved from changes in MOFs’ framework [102] or from the electrostatic interactions between adsorbate molecules [103]. Theoretical approaches from thermodynamic viewpoints support that the step likely associates with the structural transition between *np* and *lp* forms [99]. As a result, the structural flexibility and breathing behaviors are characterized with the presence of the step in the adsorption isotherm and are commonly noticed in MIL-53s (Al, Cr, Fe) [96,99,104]. However, this step is absent from the CO_2_ adsorption isotherm of MIL-47, possibly due to the presence of the μ_2_-oxo group of the metal center [99], yielding its rigid framework.

**Figure 14.**
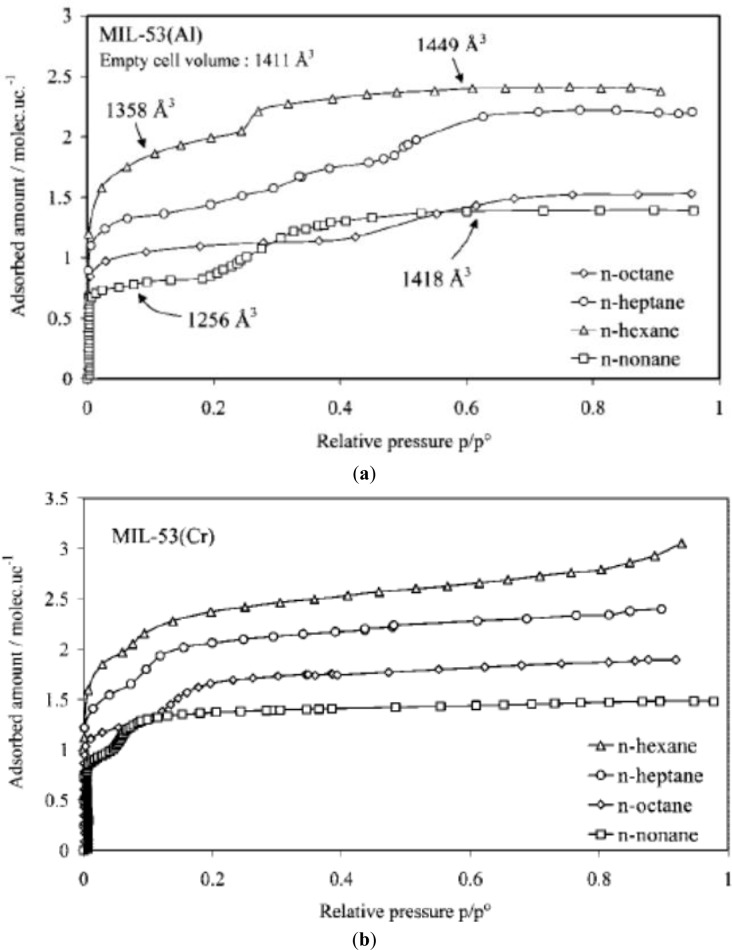
Adsorption isotherms of *n*-alkanes at 313 K. (**a**) MIL-53(Al); (**b**) MIL-53(Cr). Δ: *n*-hexane; ○: *n*-heptane; □: *n*-nonane; ⋄: *n*-octane. Reprinted with permission from [105]. Copyright 2008 American Chemical Society.

More complex breathing behaviors can be observed from the adsorption of long-chain alkanes on MIL-53s [101,105,106]. For MIL-53(Al, Cr), the stepped adsorption isotherms can be observed when propane or longer alkanes are used as adsorbates, shown in Figure 14. The occurrence of stepped adsorption isotherm shifts towards a higher relative pressure when Al is the metal node as well as longer alkanes. It was speculated that the presence of stepped isotherms was caused by host-guest interaction, entropic effects, and confinement effects.

**Figure 15.**
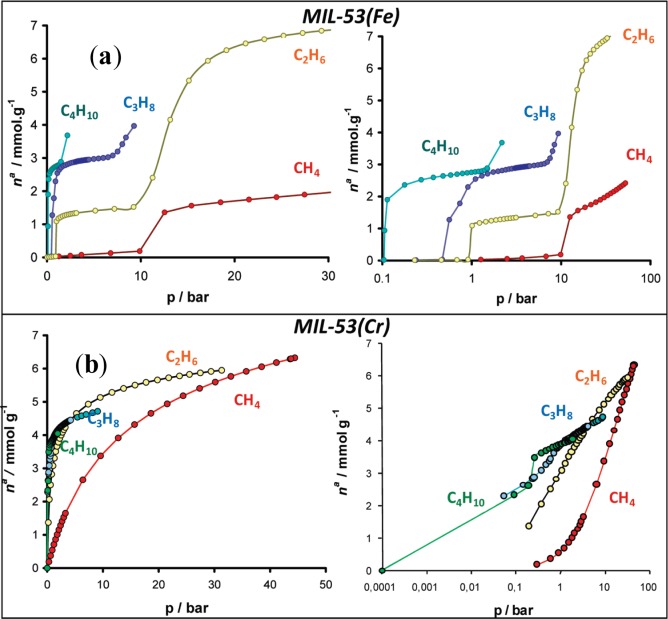
Adsorption isotherms of C1-C4 alkanes at 303 K. (**a**) MIL-53(Fe); (**b**) MIL-53(Cr). Reprinted with permission from [106]. Copyright 2009 American Chemical Society.

**Figure 16.**
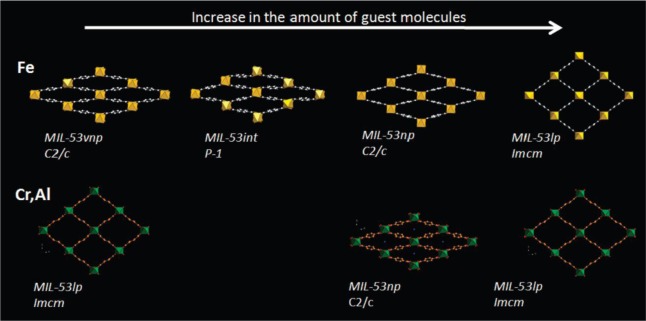
Schematic diagram of structural evolutions of MIL-53(Fe) and MIL-53(Al, Cr) upon adsorption of *n*-alkanes. Reprinted with permission from [106]. Copyright 2009 American Chemical Society.

Furthermore, shown in Figures 13 and 15, the absence of stepped adsorption isotherms when methane and ethane were used as the adsorbates suggested that the adsorption enthalpy of these adsorbates cannot overcome the threshold of adsorption enthalpy above which the breathing occurs (−20 kJ mol^−1^). In contrast, MIL-53(Fe, Sc) exhibits stepped isotherms for all tested linear alkanes, including methane and ethane. The adsorption isotherms exhibit multi-steps at various relative pressures, which have been attributed to the existence of *four* discrete pore openings changing from very narrow pore (*vnp*) → intermediate pore (*int*) → narrow pore (*np*) → large pore (*lp*). The schematic diagram is shown in Figure 16. It is noted that the stepped adsorption isotherms are absent in any hydrocarbon adsorption of MIL-47(V), further confirming the rigidity of its framework [107].

### 3.2. X-ray Diffraction

In addition to gas adsorption measurements, the structural transition associating with the breathing behavior can be characterized using *in situ* X-ray diffraction [26,27,105,106]. From powder XRD patterns (PXRDs), the crystallographic refinement of MIL-53(Cr) crystals, under the hydration-dehydration cycle, yield the unit cell dimensions of MIL-53*as* (Cr) are as follows: orthorhombic system, *Pnam*, *a* = 17.340(1), *b* = 12.178(1), *c* = 6.822(1) Å, *V* = 1440.6(1) Å^3^; for MIL-53*ht* (Cr), orthorhombic system, *Imcm*, *a* = 16.733(1), *b* = 13.038(1), *c* = 6.812(1) Å, *V* = 1486.2(2) Å^3^; for MIL-53*lt* (Cr), monoclinic system, *C*2/*c*, *a* = 19.685(4), *b* = 7.849(1), *c* = 6.782(1) Å, *V* = 1012.8(1) Å^3^, β = 104.90(2)° [108]. The result clearly shows the volume contraction and expansion during the breathing cycle while using heat as the external stimulus. Similarly, the structural transitions of MIL-53(Al, Fe) can be observed using the same technique [26,109]. The structural transition upon adsorptions of CO_2_ was also analyzed using *in situ* PXRD patterns as: MIL-53-CO_2_ (Cr): monoclinic system, *C*2/*c*, *a* = 19.713(1), *b* = 8.310(1), *c* = 6.806(1) Å, *V* = 1072.5(1) Å^3^, β = 105.85(2)° [27]. Structural transitions were also analyzed for MIL-53s upon adsorptions of hydrocarbons [101,105,106]. Analyses of unit cell parameters of MIL-53 (Al, Fe, Cr, Ga, Sc) upon applying various external stimuli are summarized in Table 3.

### 3.3. ^129^Xe Nuclear Magnetic Resonance

**Figure 17.**
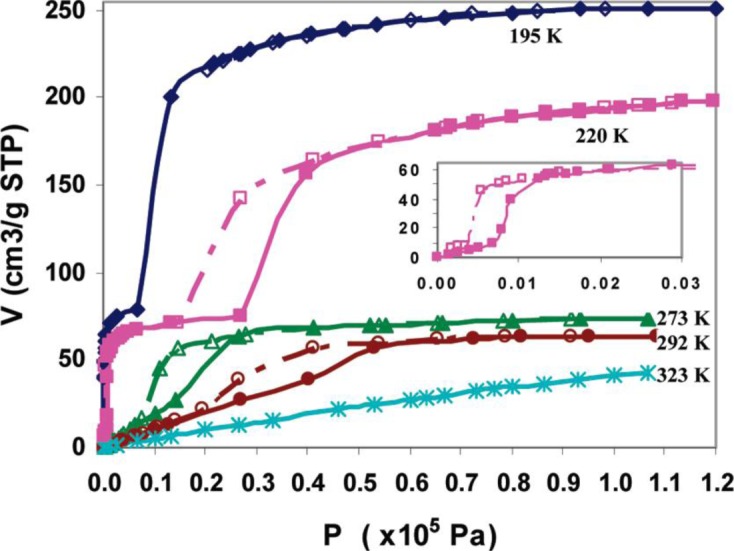
Xe adsorption (solid symbols) and desorption (open symbols) isotherms measured at 195 (lozenges), 220 (squares), 273 (triangles), 292 (circles), and 323 K (stars). Inset: Low-pressure region of the isotherm at 220 K. Reprinted with permission from [110]. Copyright 2010 American Chemical Society.

Xenon adsorption studies combined with ^129^Xe NMR spectroscopy are favorable methods for characterizations of porosity and framework flexibility of MOFs, due to the high polarizability of Xe atoms upon interacting with its environment [90,104,110]. For MIL-53(Al), adsorption isotherms of Xe below 300 K show steps and hysteresis loops, implying the structural transition from *lp* to *np* forms, shown in Figure 17.

**Figure 18.**
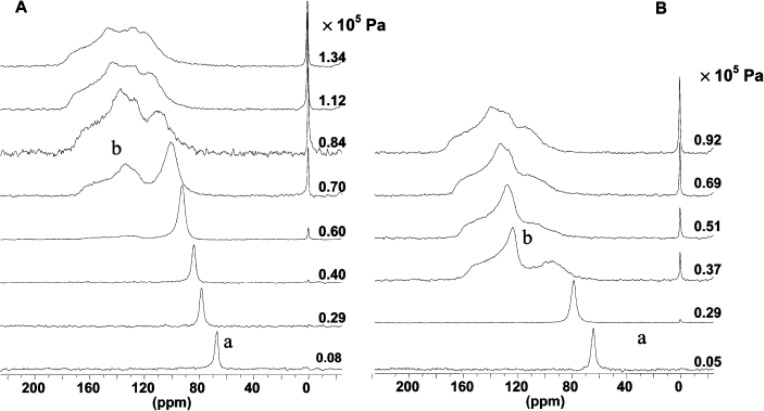
^129^Xe NMR spectra measured under various xenon pressures. (**A**) Adsorption; (**B**) Desorption. Reprinted with permission from [110]. Copyright 2010 American Chemical Society.

^129^Xe NMR spectra show a single isotropic line at the chemical shift range of 60–70 ppm (region a) under low pressure in Figure 18. The presence of isotropic line indicates the framework adopts the *lp* form. As the adsorbed Xe quantity increases, the single isotropic line exhibits higher chemical shift and less intensity. Upon reaching a threshold pressure, a reversible anisotropic line with the chemical shift range of 120–160 ppm (region b) appears, indicating the framework becomes the *np* form. The structural transition could be induced by the interaction between Xe atoms and organic parts of MOFs, yielding the rotation of phenyl rings during shrinkage. Detailed NMR analyses indicate that the extent of structural transition is not complete at room temperature, comparable to the “forceps” effect observed in MIL-53(Fe) [121]. ^129^Xe NMR technique was used to characterize the framework flexibility of DUT-8(Ni), a MOF with Ni_2_(2,6-NDC)_2_(DABCO) (DUT = Dresden University of Technology, 2,6-NDC = 2,6-naphthalenedicarboxylate, DABCO = 1,4-diazabicyclo[2.2.2]octane) [122].

### 3.4. Electron Paramagnetic Resonance (EPR) Using Nitroxide as Probes

Recently, Bagryanskaya’s group developed an alternative approach to elucidating the *generic* breathing behavior of MIL-53(Al) using electron paramagnetic resonance (EPR) [123]. A very low concentration of (2,2,6,6-tetramethylpiperidin-1-yl)oxyl (TEMPO) was introduced as the probe (1 molecule/1,000 unit cells). Its tumbling correlation times (*τ*_c_) were analyzed at various temperatures, showing that the probe molecules become immobile as the framework turns into the *np* form at 80 K. The probe molecules become freely rotating as the framework changes into the *lp* form at room temperature. The obtained reversible breathing behavior of MIL-53(Al) was comparable with those obtained using aforementioned techniques.

## 4. Thermodynamic Viewpoints

Adsorptions of various adsorbates (H_2_O, CO_2_, Xe, and *n*-alkanes) induce framework structural transitions due to adsorbate–adsorbent interactions, which is recognized as the breathing effect [124]. The striking breathing phenomena of MOFs lead to eminent steps and hysteresis in adsorption and desorption isotherms. Researchers also speculate that rationales of this unprecedented behavior via thermodynamic viewpoints can unveil strategies to “tailor” the MOFs’ gas sorption capacity.

Coudert *et al.*, recently developed thermodynamic viewpoints on framework transitions [125] in which they proposed that framework transitions are determined by five parameters: the free energy difference between the empty host structures (**1**, Δ*F*_host_), the pore volumes (**2**, *V_P_*^(1)^, and *V_P_*^(2)^), and the Henry constants (**2**, *K*_1_, and *K*_2_). These parameters consist of the widely-recognized osmotic potential ensemble model:

$$\Delta \Omega_{os}(P)=\Delta F_{host}-RT\rho \left[V_{P}^{\left(2\right)}\text{ ln}\left(1+\frac{K_{2}P}{\rho V_{P}^{\left(2\right)}}\right)-V_{P}^{\left(1\right)}\text{ ln}\left(1+\frac{K_{1}P}{\rho V_{P}^{\left(1\right)}}\right)\right]$$ (1)

whereas ∆Ω*_os_*(*P*) means the osmotic potential of the different solid phases during the guest-induced structural transitions of the host material.

**Figure 19.**
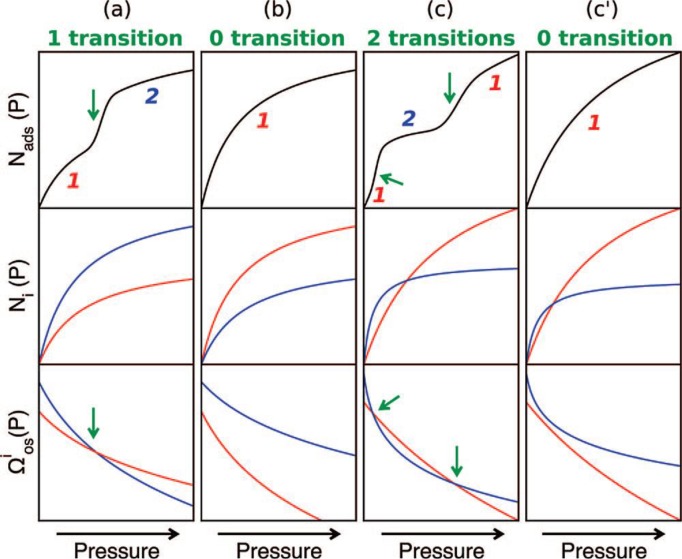
The four potential scenarios of adsorbents exhibiting the structural transition between Phases **1** (red) and **2** (blue), using osmotic potential ensemble model. (**Top**) the adsorption isotherms; (**Middle**) the Langmuir isotherms of each phase in respective scenario; (**Bottom**) the osmotic potential of each phase. Green arrows indicate the occurrence of structural transitions. Reprinted with permission from [125]. Copyright 2008 American Chemical Society.

The guest-host interaction of CO_2_ adsorption evolves from the quadrupole moment of CO_2_ molecules. The CO_2_ adsorption on MIL-53(Al, Cr) exhibits two transition steps, changing from *lp* to *np* at low pressure (0.3 bar) and from *np* to *lp* at high pressure (5 bar). At low pressure region, the free energy difference was estimated about 2.5 kJ/mole. The energy barrier of the first transition is very comparable with *kT* at room temperature. The energy barrier of the second framework transition is about 20 kJ/mole at high pressure. This framework transition relies on: (1) the affinity between adsorbate-adsorbent; (2) pore volumes; and (3) the free energy difference between the narrow-pore (*np*) and the large-pore (*lp*) states. Using the model, the authors predicted four scenarios shown in Figure 19: (a) SCSC single transition $\left(V_{P}^{\left(2\right)}>V_{P}^{\left(1\right)}\right)$: unit cell volume expands as the pressure increases; (b) no framework transition $\left(V_{P}^{\left(1\right)}>V_{P}^{\left(2\right)}\text{ and }K_{1}>K_{2}\right)$: as the empty structure is the most stable phase; (c) SCSC double transition $\left(V_{P}^{\left(1\right)}>V_{P}^{\left(2\right)}\text{ and }K_{2}\gg K_{1}\right)$: unit cell volume contracts at the low pressure and expands at the high pressure region; (d) no framework transition $\left(V_{P}^{\left(1\right)}>V_{P}^{\left(2\right)}\text{ and }K_{2}\geq K_{1}\right)$: as van der Waals force interaction is too weak to offset the free energy of framework transition (2–6 kJ/mol). The model successfully explains the presence of dual framework transitions of MIL-53(Al, Cr) upon the CO_2_ adsorption, and the absence of framework transition upon CH_4_ adsorption [125].

For MIL-53, where the *lp* form is intrinsically more stable than the *np* form at room temperature, it was predicted that either the occurrence of two structural transitions upon gas adsorption or the absence of any transition is determined by a balance between intrinsic stability of the crystal structures, adsorption affinities, and accessible volume. With respect to alkanes adsorption of MIL-53, adsorption isotherms exhibit that the variation of pore volume for both forms is small [126] and the main factor is resulted from the change in adsorption affinities of different guests [126].

**Figure 20.**
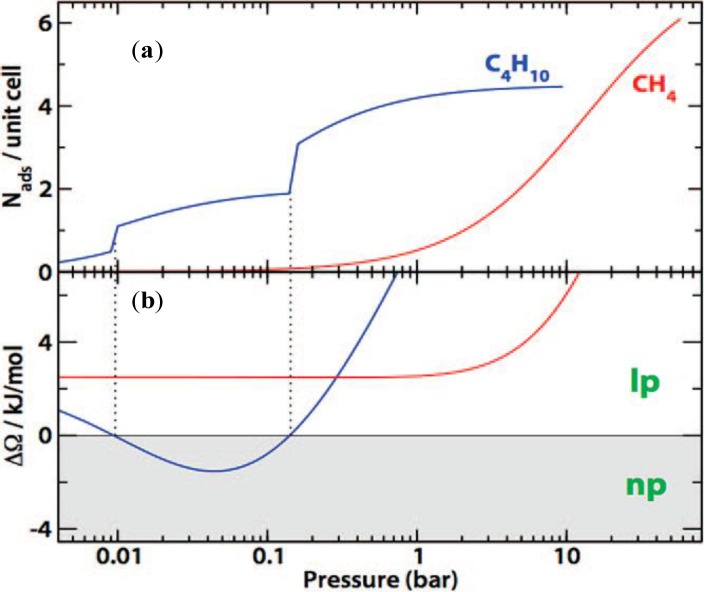
(**a**) Adsorption isotherms of CH_4_ (red) and C_4_H_10_ (blue) in MIL-53(Cr), in a Langmuir model; (**b**) the osmotic potential change during the structural transition between the *lp* and *np* forms. Vertical dotted lines indicate the structural transition induced by C_4_H_10_ molecules. Reprinted with permission from [126]. Copyright 2009 American Chemical Society.

The absence of framework transition upon CH_4_ adsorption was explicitly predicted using the concept of relative affinity, *K*_np_/*K*_lp_, which is a function of pore volumes and the free energy difference between *np* and *lp* states [126]. As the relative affinity is much larger than unity, the osmotic potential difference switches sign twice, leading to the presence of double framework transitions between *lp* and *np* forms upon long-chain hydrocarbon adsorptions, shown in Figure 20. Due to the low relative affinity of CH_4_ (almost equal to unity), the *lp* form is thermodynamically stable, yielding no step of its adsorption isotherm [126].

**Figure 21.**
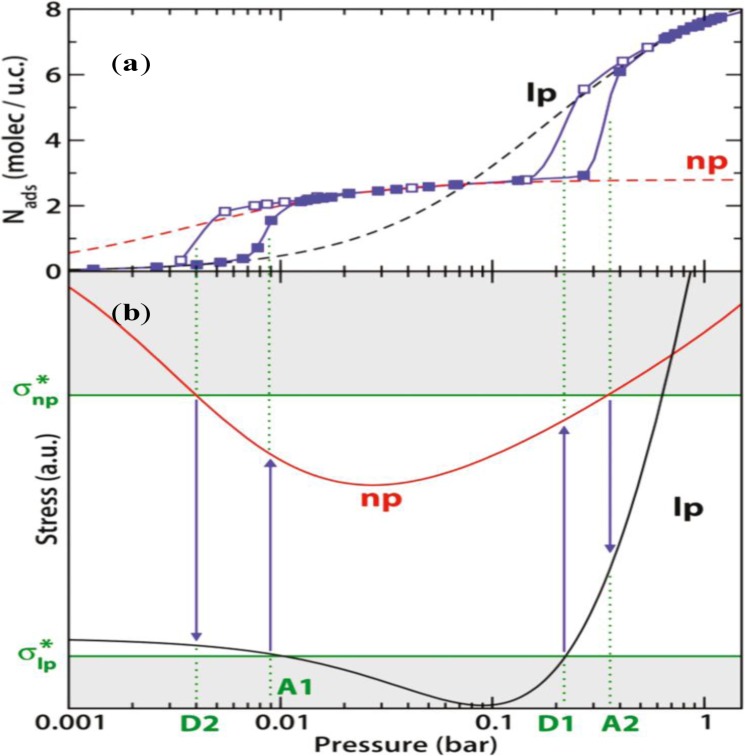
(**a**) Experimental adsorption (filled) and desorption (empty) isotherms of Xe in MIL-53(Al) at 220 K (in blue), and the fitting of Langmuir isotherms using the *lp* (black dash) and *np* (red dash) forms, respectively; (**b**) simulated structural transition stresses of the *lp* (black) and *np* (red) forms. σ*_np_ and σ*_lp_ denote the critical stresses of the structural transitions upon adsorption (A1 and A2) and desorption (D1 and D2), respectively. Reprinted with permission from [128]. Copyright 2010 American Chemical Society.

The affinity between adsorbate (Xe)-adsorbent (MIL-53(Al)) was measured using the Henry’s constant (*K*_H_) at *np* and *lp* forms, which in turn was used to estimate the adsorption stress (σ_s_) during the structural transition [127,128]. At the low pressure region when adsorbate molecules are introduced, the framework starts contracting, yielding decreasing framework stress. Once the critical stress is reached $\left(\sigma_{lp}^{*}\right)$, the *lp* form becomes unstable and the framework turns into the *np* form. During the period, MOFs are experiencing the “abnormal breathing” behavior—the sample contracts while inhaling. This results in a sharp uptake step on the adsorption isotherm; the sample volume decreases by 40% (line A1 in Figure 21). As the pressure increases, the *np*-form framework starts expanding, which yields the increasing framework stress. Once the critical stress is reached $\left(\sigma_{np}^{*}\right)$, the *np* form becomes unstable. The framework turns into the *lp* form, during which the sample expands while “inhaling”. The sample volume increases by 40%, displayed by the second step on the adsorption isotherm (line A2). As the adsorption occurs further in the *lp* phase, the solvation pressure increases, causing the elastic expansion of sample, which remains stable [128]. This stress-based model implies that two distinctive states of framework exist, which cannot explain the coexistence of *np* and *lp* forms during the contracting state [110,127].

From molecular dynamics (MD) simulations, translational (τ) and orientational (*S*) orders of confined CO_2_ molecules were used to monitor the framework transition. As the structural transition (*lp* → *np* form) upon the adsorption occurs at low pressure region, the orientational order of CO_2_ increased drastically due to the confinement of *np* form. Similar change can be noticed at the translation order loss along the channel direction, indicating the rearrangement of CO_2_ molecules while the framework contracts. It is suggested that the heat of adsorption via the guest-host interaction can compensate for the entropy loss of confined CO_2_ molecules, making the framework transition becomes an enthalpy-driven step [127].

**Figure 22.**
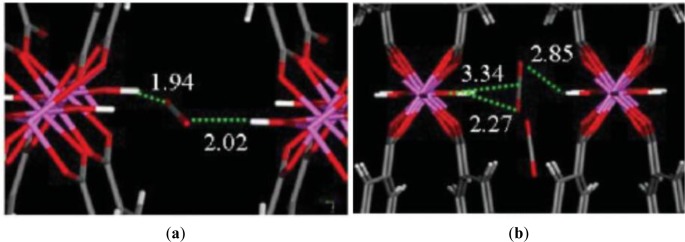
Simulation of CO_2_ arrangement of the adsorption on MIL-53(Al). (**a**) at the initial stage of loading in MIL-53*np* (Al); (**b**) at the high stage of loading in MIL-53*lp* (Al). Reprinted with permission from [130]. Copyright 2008 American Chemical Society.

At the low pressure region in Figure 22a, it was shown that a dual-interaction between the O atoms of CO_2_ and two hydrogen atoms of μ_2_ hydroxyl groups on the opposing pore walls presents in both the *lp* and *np* forms of MIL-53(Al) [129,130]. The distances of O_CO2_-H_μ2-OH_ are comparable with the hydrogen bonds of clathrated water interacting with the carboxylate groups in MIL-53*as* (Al) [26]. While the adsorption takes place at high pressure region (>5 bars), in Figure 22b, all four of the hydroxyl groups present are involved in forming this dual-interaction. In addition, the adsorbate-adsorbate interaction becomes more prominent, resulting in the formation the single-interaction between CO_2_ adsorbate and both organic linker and μ_2_ hydroxyl group, which subsequently leads to a more open porosity. This initiates the structural transition from the *np* form to the *lp* form. As the pores are further filled, the adsorbate molecules mainly interacts with the hydrogen atoms on the organic groups, as well as the inorganic part [129].

**Figure 23.**
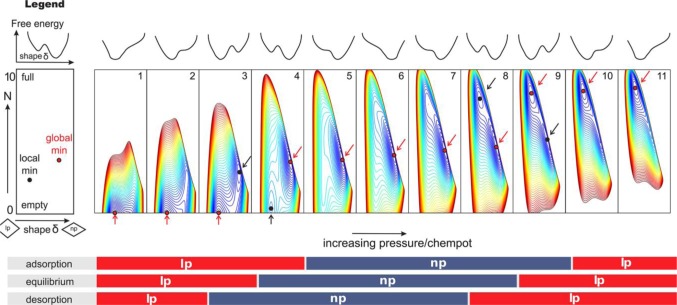
Free energy simulation of CO_2_ adsorption on MIL-53(Cr) at 300 K under various pressure ranges. The blue-colored contour represents the lowest free energy, while the red-colored contour means the highest free energy. The under panel shows the expected framework structure during adsorption, equilibrium and desorption processes. Reprinted with permission from [132]. Copyright 2013 American Chemical Society.

An inflection point was observed in the simulated energetic profile during the framework transition, indicating that an energetic unfavorable intermediate state exists during the framework transition of CO_2_ adsorption on MIL-53(Cr). This observation implies that the framework transition could be a *progressive type* instead of a distinct one, meaning that some part of pores are open while the remaining are closed [131]. Shown in Figure 23, the free energy model that incorporates host free energy, guest-guest interactions, and guest-host interaction is introduced successfully to reproduce the CO_2_ and CH_4_ adsorption isotherms of MIL-53(Cr) [132]. Under the low and high pressure regions, a global minimum of free energy is observed (red dots), indicating the *lp* form is the single stable framework structure (plots 1, 2, 10, and 11). Upon changing the pressure, a local minimum of free energy (black dots) presents, showing that the *np* form coexists with the *lp* form (plots 3, 4, 8, and 9). Further changing the gas pressure leads to the disappearance of *lp* form, and the framework becomes the pure *np* form (plots 5, 6, and 7). The model also rationalizes the framework transition depends on the type and the size of guest molecules [127,132,133]. The observations are corroborated with experimental data [110].

**Figure 24.**
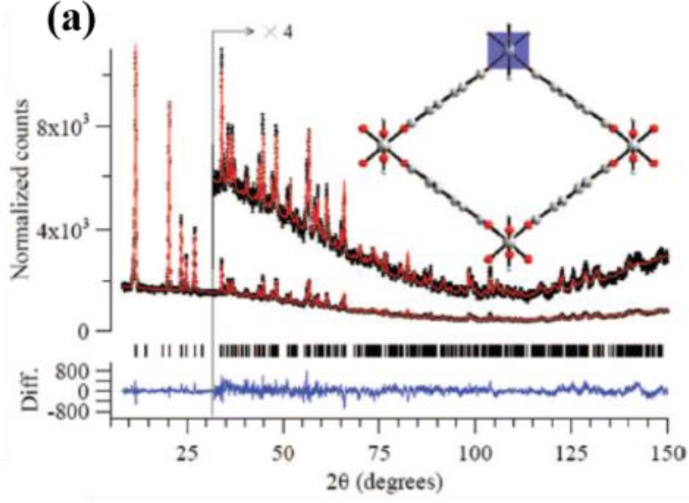

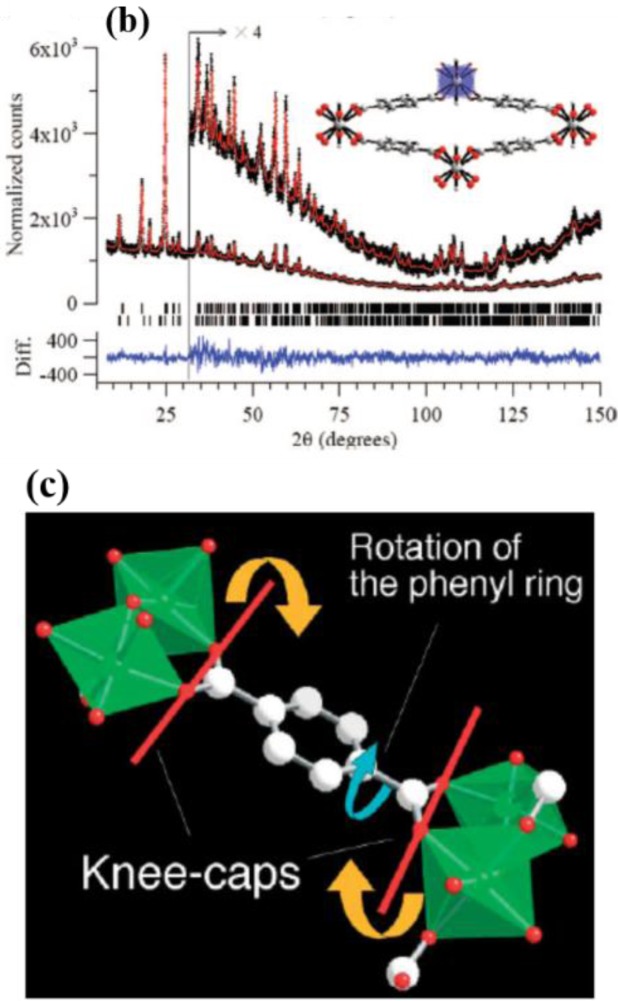
Neutron powder diffraction of MIL-53(Al) collected at (**a**) an elevated temperature (*lp* form); (**b**) room temperature (*np* form). The π–flipping of BDC ligands and the distortion of octahedral metal nodes can be visualized in the inset and (**c**). Reprinted with permission from [112]. Copyright 2008 American Chemical Society. [27]. Copyright 2007 WILEY-VCH Verlag GmbH & Co. KGaA, Weinheim.

Other than guest molecules, the framework transition can be triggered by temperature variation, leading to the dehydration-rehydration cycle. It has been shown that MIL-53 exhibits the *lp* form under high temperature (>300 K) and the *np* form under low temperature (<300 K). With the absence of van der Waals force interactions between adsorbate and adsorbent, the framework transition occurs through (1) twisted benzene groups of benzenedicarboxylate (BDC) ligands, also called as π flipping; and (2) distortion mode from the corner-sharing octahedral MO_6_ (M = Al, Cr) clusters, displayed in Figure 24 [112,134]. Similar temperature-dependent framework transition is also observed for MOF-5 [135].

## 5. Functionalization of MOFs to Modulate Breathing Behaviors

For gas storage and separation applications, it is widely recognized that the gas uptake capacity and gas adsorption selectivity are correlated with the pore size and pore shape of MOFs. Altering the pore size and pore shape of MOFs can be achieved by designing new MOFs using new organic linkers or metal centers prior to synthesis. The approach enjoys the advantage of one-pot synthesis, to tailor the pore size, adsorption affinity, and separation selectivity. However, the approach of incorporating predetermined or pre-functionalized organic ligands may be not impeccable, due to their poor stability, reactivity, and solubility under reaction conditions. This drawback has driven the needs to modify the MOF framework functionality using post synthetic modifications (PSMs) [136–141]. PSMs are chemical treatments of parent MOFs, while maintaining the structure intact. Almost all PSMs are based on amine-bearing organic linkers. Requirements for successful PSMs includes: accessible porosity or surface, available functional groups, and stability under reaction conditions and environment. The covalent PSM on MOFs was first implemented by Kim’s group, on which free pyridyl groups of organic moieties was treated [142]. There have been several excellent review articles published specifically on this topic [143], and is not detailed in this work. Other approaches adopted include using: (1) pre-functionalized organic linkers [76,115,144]; (2) mixed organic linkers [145,146]; (3) different metal nodes [147]; (4) mixed metal ions as nodes in MOFs [96].

### 5.1. Pre-Functionalized Organic Linkers

Tuning the breathing effect of MOFs has recently been achieved by designing the structure of organic linkers at the pre-synthesis stage. MIL-53 structure contains linkages of AlO_4_(OH)_2_-octahedra with 1,4-benzenedicarboxylic acid (BDC), resulting in one-dimensional channels in *c* direction. The inclusion of amine-bearing organic linkers in MIL-53s leads to the slight expansion of unit cell volume [148]. However, it exerts no change on the MOF structure nor the breathing behaviors. The expansion of unit cell volume could be originated from the occupation of amine groups inside channels. The occupation of amine groups also result in less adsorption capacity and less thermal stability, compared with non-functionalized MIL-53.

A similar effect can be observed for NH_2_-MIL-53 on the adsorption capacity of CO_2_ which decreases from 40 wt% to 30 wt%, due to the less available micropore volume. However, NH_2_-MIL-53 exhibits a higher separation factor of CO_2_ over CH_4_, due to the coupling contribution from the strong interaction between NH_2_ groups and CO_2_ molecules and the flexible framework of MIL-53 [149]. It was determined experimentally that the zero coverage adsorption enthalpy of CO_2_ is increased from 20.1 kJ/mol to 38.4 kJ/mol when MIL-53 framework is decorated with amino groups.

Amino groups of NH_2_-MOFs are considered as tags for creating versatile functionalities using PSMs [136,150]. In addition to the amino group, various pre-functionalized organic linkers are adopted in synthesizing Al- and Fe-based MIL-53s [111,115]. Stock’s group solvothermally synthesized five flexible functionalized MIL-53(Al) using BDC-X as linkers, whereas BDC = 1,4-benzenedicarboxy-late; X = –Cl, –Br, –CH_3_, –NO_2_, and –(OH)_2_. The breathing behaviors of the MOFs were altered based on the appended functional groups. During the hydration-dehydration cycle, all materials demonstrated the *np*-to-*lp* transition at various temperatures, in which Cl-MIL-53(Al) occurs at 110 °C, Br-MIL-53(Al) is at 80 °C, CH_3_-MIL-53(Al) is at 120 °C, and NO_2_-MIL-53(Al) is at 130 °C. This phenomenon was attributed to the hydrogen bonding interactions between the adsorbate and absorbents. However, the *lp* form was originally observed in (OH)_2_-MIL-53(Al) below 120 °C and was transformed into a pure *np* form above 140 °C, due to the removal of water molecules. This work has recently been extended to investigate the effect of fluorinated BDC on the sorption capacity of F-MIL-47 and F-MIL-53(Al) [151,152]. F-MIL-47 exhibits better *n*-hexane uptake capacity than its parent materials, possibly due to the enhanced hydrophobicity of its framework. The F-MIL-53(Al) shows the decreased *n*-hexane adsorption capacity, possibly originated from its framework rigidity.

**Figure 25.**
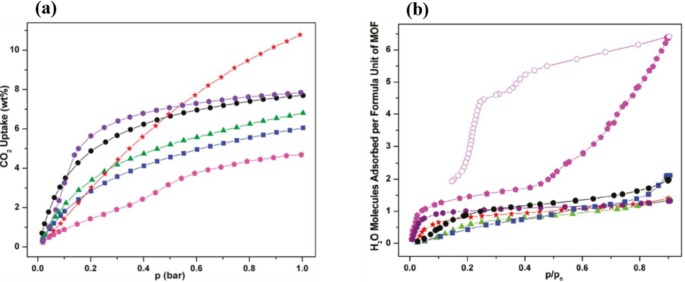
Adsorption isotherms of functionalized MIL-53s: (**a**) CO_2_; (**b**) H_2_O. Symbols: (Image036): Cl-MIL-53(Al); (Image037): Br-MIL-53(Al); (Image038): CH_3_-MIL-53(Al); (Image039): NO_2_-MIL-53(Al); (Image040): (OH)_2_-MIL-53(Al); (Image041): NH_2_-MIL-53(Al). Reprinted with permission from [111]. Copyright 2011 American Chemical Society.

The functionalized MIL-53(Al)s showed smaller accessible micropore volumes compared with their parent material, due to the occupancy of functional groups inside the one-dimensional channels. The accessible micropore volumes demonstrate the decreasing trend in the following order: NO_2_-MIL-53(Al) > Cl-MIL-53(Al) = CH_3_-MIL-53(Al) > Br-MIL-53(Al) > (OH)_2_-MIL-53(Al), due to the steric hindrance of functional groups. Their CO_2_ adsorption capacities exhibited the similar trend; however, all of them were smaller than NH_2_-MIL-53(Al), shown in Figure 25. This trend is owing to the availability and affinity of amino groups towards CO_2_ molecules. It is noted that the CO_2_ adsorption capacity of –NO_2_ is higher than that of NH_2_-MIL-53(Al) when CO_2_ pressure was above 1 bar, due to the presence of *np* form of NH_2_-MIL-53(Al).

**Figure 26.**
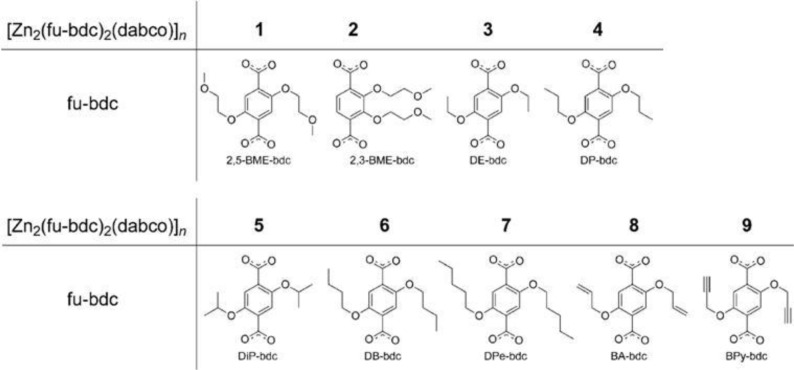
Chemical structure of derivatives of 2,3-dihydroxyl-BDC and 2,5-dihydroxyl-BDC. Reprinted with permission from [76]. Copyright 2012 American Chemical Society.

**Figure 27.**
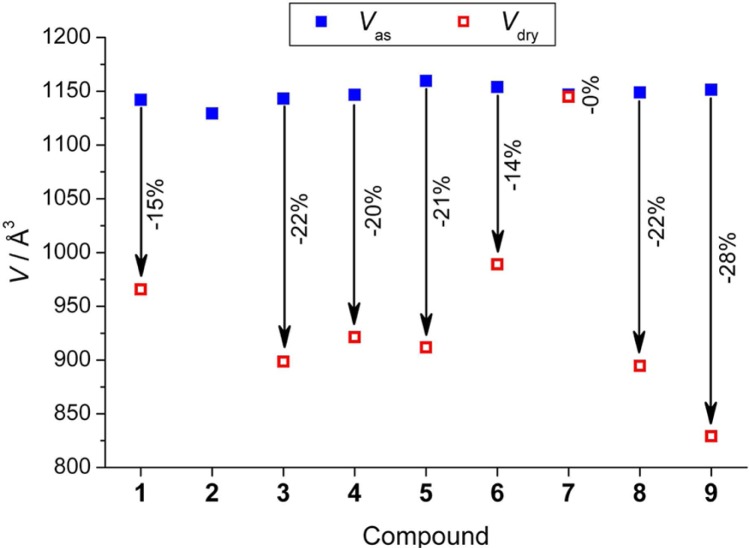
The volume contraction of various functionalized MOFs [Zn_2_(functionalized-BDC)_2_(DABCO)]_n_. Reprinted with permission from [76]. Copyright 2012 American Chemical Society.

Furthermore, several derivatives of 1,4-benzenedicarboxylic acid (BDC)-based organic linkers with flexible side chains were prepared through esterification reactions, forming 2,3-dihydroxyl-BDC and 2,5-dihydroxyl-BDC [76]. The breathing behavior of the pillar-layered MOFs [Zn_2_(functionalized-BDC)_2_ (DABCO)]_n_ was tailored with various functional groups, illustrated in Figure 26. The dangling substituted groups form stronger attraction forces (van der Waal, hydrogen bonding, or π–π interactions), yielding more contraction of unit cell volume, ranging from 86% to 72% (Figure 27). However, if the linker is substituted at Positions 2 and 3 of BDC ligands, the frameworks become much rigid, compared with those formed from 2,5-di-substituted ones.

**Figure 28.**
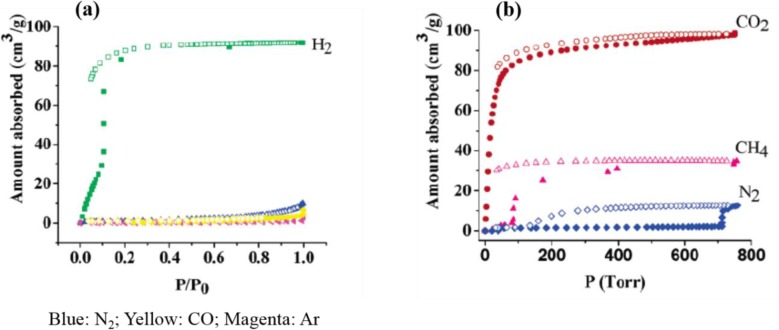
MOF using M_2_(COO)_4_ as the metal node, and 4,4′-Bpe as the organic linker. The MOF exhibits selective adsorptions towards (**a**) H_2_ and (**b**) CO_2_. Reprinted with permission from [154]. Copyright 2007 American Chemical Society.

**Figure 29.**
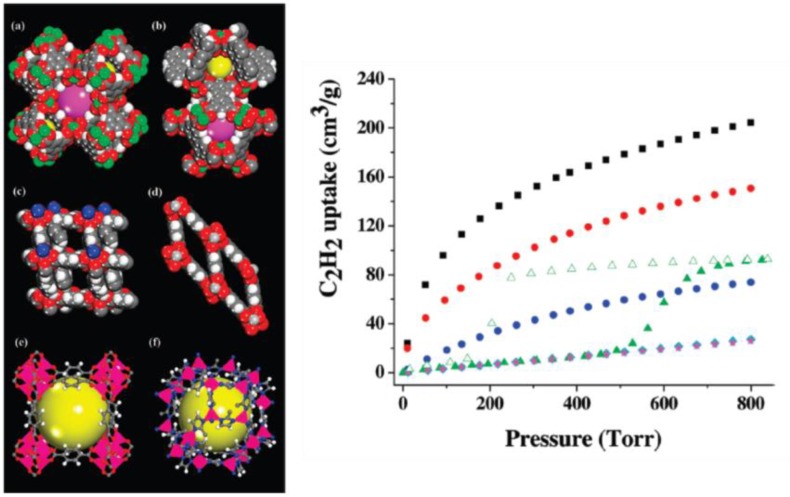
(**Left**) crystal structures of (**a**) HKUST-1; (**b**) MOF-505; (**c**) MOF-508; (**d**) MIL-53; (**e**) MOF-5; and (**f**) ZIF-8, in which (**a**) and (**b**) show open Cu^2+^ sites (green). (**Right**) the C_2_H_2_ adsorption isotherms of several MOFs, HKUST-1 (black); MOF-505 (red); MOF-508 (green); MIL-53 (blue); MOF-5 (cyan); and ZIF-8 (magenta). Reprinted with permission from [155]. Copyright 2009 American Chemical Society.

The chemical treatment can decorate the channels of MOF frameworks with controlled and various functionalities. The approach is very suitable for applications in molecule recognition via several types of interaction, such as van der Waals force, hydrogen bonds between MOF frameworks and substrates [153]. The authors proposed three approaches to tune the functionalities of MOFs in differential microenvironment: (1) the formation of cubic nets as interpenetrating frameworks, by combining paddle-wheel clusters M_2_(COO)_4_ and dicarboxylic acid (BDC), and pillar bi-dentate organic linkers. By changing the organic linker from 4,4-bipyridine (4,4′-Bipy) to *trans*-bis(4-pyridyl)-ethylene (4,4′-Bpe), the resulting MOFs exhibit selective sorption towards H_2_ and CO_2_ and selective separation of linear and branched isomers (Figure 28) [154]; (2) immobilization of open metal centers (Cu^2+^) within channels showing extraordinarily high acetylene storage at room temperature, making the transportation of high density acetylene storage for industrial processes (Figure 29) [155]; (3) immobilization of open metal centers using coordinating ligands, such as Schiff base, for separation.

### 5.2. Mixed Organic Linkers

**Figure 30.**
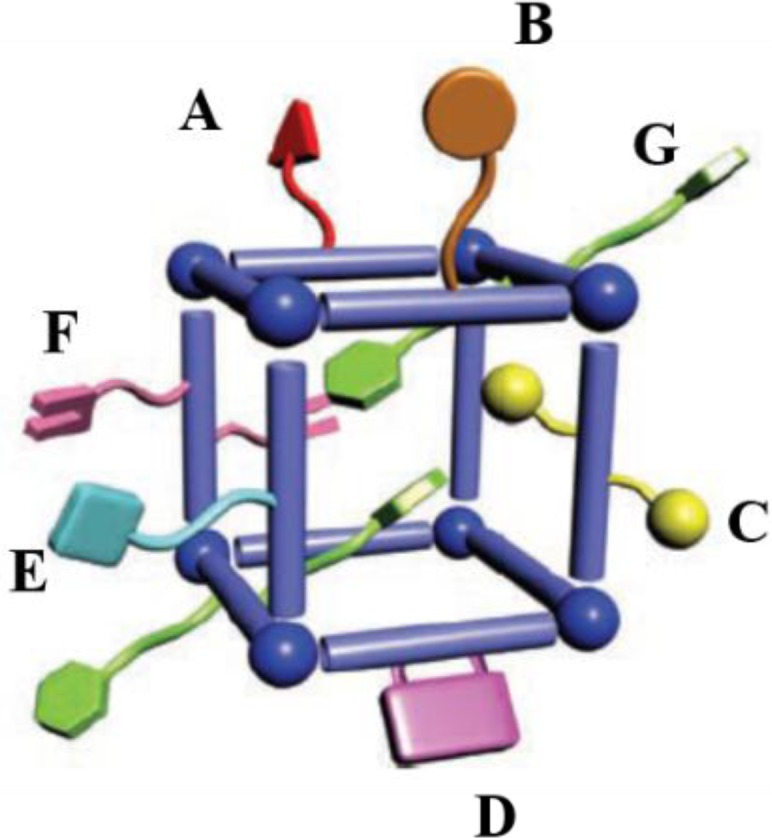
MTV-MOF-5 equipped with distinct functionalities in one phase. Reprinted with permission from [144]. Copyright 2010, American Association for the Advancement of Science.

**Figure 31.**
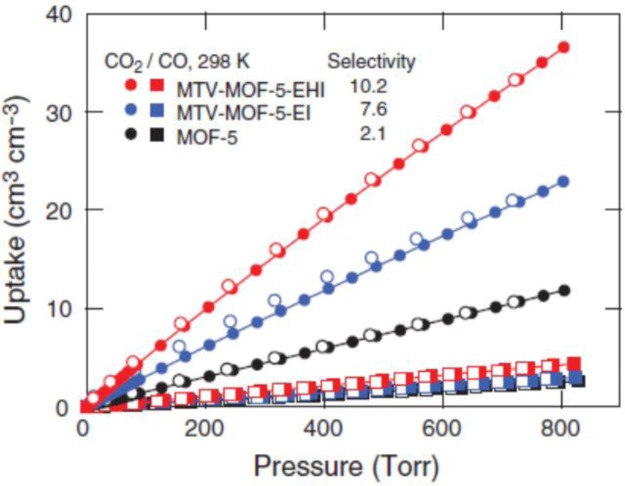
Several MTV-MOF-5s exhibit enhanced CO_2_/CO selectivity. **E**: NO_2_-BDC; **H**: (C_3_H_5_O)_2_-BDC; **I**: (C_7_H_7_O)_2_-BDC. Reprinted with permission from [144]. Copyright 2010, American Association for the Advancement of Science.

The breathing behavior, adsorption capacity, selectivity, and thermo-mechanical property of MOFs can be tuned by mixing various organic linkers. Yaghi’s group synthesized 18 one-phase multivariate (MTV) MOF-5s that contains distinct functionalities, using 8 types of functionalized 1,4-benzenedicarboxylate (BDC) as organic linkers, shown in Figure 30 [144]. Compared with un-functionalized MOF-5, the resulted MOFs exhibits enhanced H_2_ uptake capacities (84%, maximum), and improved CO_2_/CO selectivity (400%), shown in Figure 31. Similarly, an improved CO_2_/N_2_ selectivity was reported by Costantino’s group using mixed organic linkers, in which a water-stable isoreticular phosphonate MOFs are successfully formed [156]. Farrusseng’s group synthesized a series of MIL-53s using various ratio of BDC and amino-substituted BDC [124]. The resulted MOFs show smaller micropore volume as the adopted functionalized BDC content is increased. The higher content of amino groups leads to higher Henry constants, yielding lower *lp-*to-*np* phase transition pressure and higher *np-*to-*lp* phase transition pressure, as well as the gas adsorption capacity. It is noted that the altered phase-transition temperature of MOFs synthesized from the mixed-linker approach also affects their thermo-mechanical properties, recently reported by Fischer’s group, depending on the chain length and the hydrophobicity of the functionalized groups [146].

### 5.3. Different Metal Nodes

**Figure 32.**
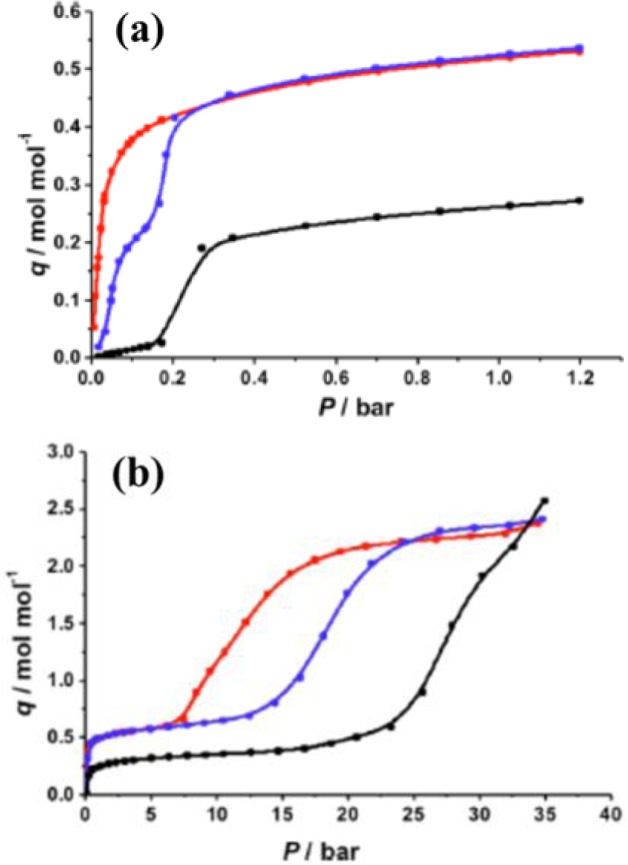
CO_2_ adsorption isotherms at 273 K. (**a**) 120 kPa; (**b**) 3.5 MPa. Red: NH_2_-MIL-53(Al); Blue: NH_2_-MIL-53(Ga); Black: NH_2_-MIL-53(In). Reprinted with permission from [147]. Copyright 2012 American Chemical Society.

Gascon’s group investigated the effect of metal node on the breathing behaviors of MOFs [147]. In their study, Al, Ga, and In were successfully used as various metal nodes to synthesize amino-functionalized MIL-53s. Measurements of CO_2_ adsorption on these MOFs showed that NH_2_-MIL-53(Al) displayed a Langmuir isotherm (Type I) while its Ga- and In-derivatives exhibited stepped isotherms respectively, shown in Figure 32. The presence of stepped isotherm was attributed to the existence of observable *vnp* form in NH_2_-MIL-53(Ga) and NH_2_-MIL-53(In), which are oriented from the electropositivity of the metals. The distance between amine groups of organic liners and O_μ__2_ of a metal node decreases in the order of Al < Ga < In, showing a stronger acidity of μ_2_-OH in NH_2_-MIL-53(In) which in turn modulated the resulted breathing behaviors.

MIL-47 and MIL-53 are iso-structural MOFs with different metal centers. The former has V^IV^ as its metal centers, and the later has Al^III^ or Cr^III^ as its metal nodes. The existence of corner-sharing μ_2_-OH groups in MIL-53 framework contribute to it framework flexibility. However, the presence of μ_2_-oxo groups in MIL-47 framework yields the rigidity of its framework. Hence, MIL-47 only shows as a microporous material and exhibits no breathing behaviors [47,99]. How does the framework functionality affect their gas adsorption behaviors and breathing phenomena?

NH_2_-MIL-47 shows less CH_4_ and CO_2_ adsorption capacities compared with MIL-47 at 30 °C within test pressure range [157]. The declined gas adsorption capacity is owing to the presence of dangling amino-groups in the pores, yielding a decrease in the pore volume (from 0.46 mL/g to 0.40 mL/g). However, the presence of NH_2_ groups doesn’t affect significantly the affinity towards CO_2_ and CH_4_ molecules. This feature results in a similar separation factor of CO_2_ and CH_4_ between NH_2_-MIL-47 and MIL-47. The separation factor of CO_2_ and CH_4_ is defined as the ratio of Henry adsorption constants of CO_2_ over CH_4_. The absence of significant change on the CO_2_ and CH_4_ affinities could be due to the rigidity of NH_2_-MIL-47 framework. DFT analyses indicate that the adsorption of CO_2_ molecules is preferred towards NH_2_ groups in NH_2_-MIL-47, due to strong electrostatic interactions between the adsorbate carbon and the negative charge of the nitrogen. Similar results can be noticed on F-MIL-47 framework [151], and NH_2_-MOF-5 (IRMOF-3) [158].

Similarly, Loiseau’s group [119] reported the effect of metal identity on the breathing behavior of MIL-53s, in which MIL-53(Ga) exhibits the breathing behavior upon dehydration-hydration cycle, due to the presence of μ_2_-hydroxo linkages in its framework. An intermediary phase showing two types of closed channels was observed, in which one type of channels has a strong hydrogen-bond guest-host interaction, while the other type has a weak hydrogen-bond interaction. These interactions contribute to the thermal stability of *np*-form of MIL-53(Ga) and MIL-53(Fe). MIL-53(Al) demonstrates better thermal stability of *lp* framework without the presence of the intermediary *np* form. The effect of metal identity on the framework thermal stability follows: Al > Ga > Fe.

### 5.4. Mixing Metal Nodes

**Figure 33.**
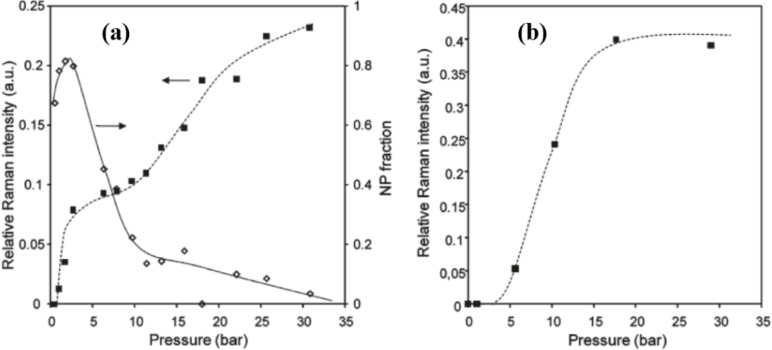
CO_2_ adsorption isotherms at 303 K. (**a**) MIL-47(V^III^); (**b**) MIL-47(V^IV^). Symbols: ■—adsorbed CO_2_; ⋄—fraction of narrow pores in MOF determined by Raman spectra. Reprinted with permission from [161]. Copyright 2011 American Chemical Society.

Tuning the breathing behavior of MOFs can be achieved via mixing cations or altering the valence of metal cations. As aforementioned, MIL-47(V^IV^) is a rigid MOF. Its framework can demonstrate the flexibility or even the breathing behavior using biphenyl-4,4′-dicarboxylate as the organic linker [159] or using high mechanical pressure [160]. Clet’s group reported to activate the flexibility of MIL-47(V) by changing the metal valence from V^IV^ to V^III^ [161]. The framework of hydrated MIL-47(V^III^) adopts the *np* form, which is in contrast to the *lp* form of MIL-47(V^IV^). The *reversible* thermal response of MIL-47(V^III^) is similar with MIL-53(Fe), following the framework transition of *np* → *cp* → *int* → *lp* as the temperature increases. The breathing behavior of MIL-47(V^III^) is exhibited by the adsorption of CO_2_, in which presents a stepped adsorption isotherm shown in Figure 33. This flexibility is owing to the presence of *μ*_2_-OH groups in MIL-47(V^III^) and is gradually diminished as the fraction of V^IV^ in MIL-47(V^III^/V^IV^) increases. The flexibility of MIL47(V^IV^) can be also induced by doping various amount of Al^III^ ions into its framework [162]. The presence of vanadyl units in pseudo-octahedral or square-pyramidal geometry might contribute to the breathing behavior of MIL-47(Al^III^/V^IV^) upon the CO_2_ adsorption.

**Figure 34.**
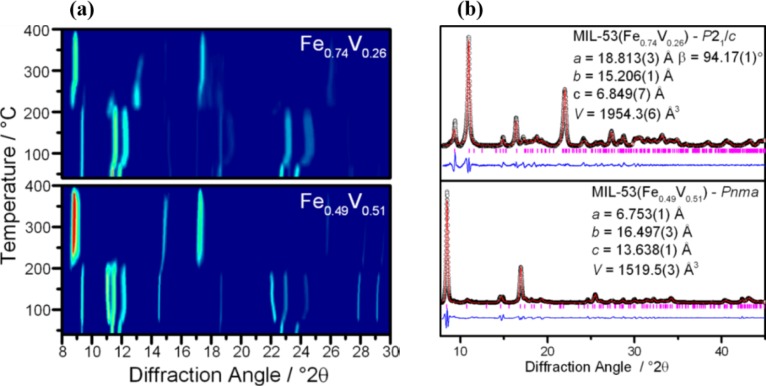
(**a**) Temperature variation of PXRD patterns of MIL-53(Fe^II^/V^III^) upon heating; (**b**) room temperature PXRD patterns of MIL-53(Fe^II^/V^III^) calcined at 573 K. Reprinted with permission from [163]. Copyright 2013 American Chemical Society.

Using a mixture of Fe-Cr as cations, Serre’s group was able to tune the breathing behaviors by taking advantage of the nature of cation ions upon the hydration-dehydration cycle [96]. While those solids are hydrated, they all adopted the *np* form; whereas MIL-53(Cr) and MIL-53(Fe) change to the *lp* form and a closed pore (*cp*) form respectively, under dehydrated conditions. This is caused by the departure of H_2_O molecules, yielding the *cp*-form structure followed by the *lp* form. However, the thermal response behavior of MIL-53(Fe^II/III^/V^III/IV^) becomes different, depending on the Fe/V ratio [163]. For Fe-rich MIL-53(Fe^II^/V^III^), its framework transforms reversibly from *np* → *int* → *lp* upon the hydration and dehydration cycle (Figure 34a). However, the framework shows irreversible transition into the *lp* form upon heating when the Fe/V ratio is 1, shown in Figure 34b. The modulation of breathing behaviors by mixing cations is also reported by Biradha’s group in {[M(L)_2_(H_2_O)_2_]·(ClO_4_)_2_·2(CH_3_OH)·2(CHCl_3_)·4(H_2_O)}*_n_* (M = Zn(II), Cd(II); L = benzene-1,3,5-triyltriisonicotinate) [164]. The MOFs using a mixture of Zn and Cd show higher N_2_ adsorption capacity compared with each parent material.

## 6. Outlooks

In addition to the dimensionality of organic ligands and metal clusters, it is clearly noticeable that those MOFs exhibiting the breathing behavior have two common features: (1) non-rigid areas, or weak points, appear in the frameworks and (2) a free space within the framework to accommodate the steric hindrance of the movements of weak points [7]. Hence, some empirical rules have been developed to predict the presence of weak points in MOFs: (1) inorganic clusters might possess a mirror plane with the organic ligands in symmetrical position; (2) O–O axes of carboxylates are perpendicular to the elongation axis. Similar requirement can be noticed very recently [159]; (3) include even cycles at the level of the cluster and/or at the level of the topology of the skeleton; (4) the ratio C/M should be ≥2, whereas C is the number of carbons of the carboxylate surrounding the cluster, and M is the number of metallic atoms within the cluster; (5) breathing effects can only occur with ditopic carboxylates, but not on MOFs with tri- or tetratopic ligands. In addition, it is reported that the metal centers must possess μ_2_-hydroxyl groups that are preferred interacting sites for adsorbates, leading to the flexibility point. The presence of μ_2_-oxo groups in MIL-47 (V^IV^) still contribute to the rigidity of framework even though all above conditions are satisfied [161]. Furthermore, more flexible MOFs showing phenomenal breathing behaviors are synthesized via tritopic ligands [81,165] or tetratopic ligands [11,166]. The flexibility is evolved from even cycles in their skeleton topologies, despite of forming odd cycles at the cluster level. An alternative approach is to create additional “kneecaps” (rotating moiety) within a bulky organic ligand (semi-rigid ligand), shown in Scheme I. Upon exposing to external stimuli, the twisted organic ligands lead to the framework flexibility.

Recently, Huang *et al*. [4] reported a breathing MOFs [Zn_3_(OH)_2_(btca)_2_]·DMF·4H_2_O (H_2_btca = benzotriazole-5-carboxylic acid). The detail structure analysis indicates that all above conditions are satisfied; however an isostructural MOF [Co_3_(OH)_2_(btca)_2_]·3.7H_2_O doesn’t exhibit the breathing effect upon dehydration [167], suggesting the breathing phenomenon of MOFs is a synergetic effect from the coordination of metal centers, and the strength of host–guest and guest–guest interactions. The author claimed that the formation of zigzag rod structure in parallel with the channel direction should be taken into the consideration, which was supported by O’Keeffe’s prediction [63].

## 7. Conclusions

Metal-organic frameworks (MOFs) have gained intense research attention in applications of gas adsorption/storage, gas separation, catalysts for fine chemicals, and biomedical imaging, among others, due to their unprecedented large surface area, tunable framework functionality, decent thermal stability, and the most striking phenomenon of framework flexibility (breathing). The breathing behaviors are characterized using *in situ* X-ray diffraction, gas adsorption, and nuclear magnetic resonance spectroscopy upon the exposure of various external stimuli, such as heat, pressure, and gases. Theoretical calculations are used to elucidate the principles of breathing behaviors using density function theory (DFT) and molecular dynamic simulations. Both approaches indicate that the breathing behaviors of MOFs evolve from synergetic effects of coordination symmetry of metal nodes and rotatable axis of organic ligands, as well as strong guest-host interactions. Post-synthetic modifications and pre-functionalized organic ligands are accommodated to rationally design the extent of breathing behaviors. Syntheses of MOFs are mainly conducted in solvothermal conditions, in which the cost and recovery of organic solvents becomes the major hurdle for large-scale production. Exploring viable hydrothermal synthesis conditions of MOFs are commensurate with the design strategies towards the breathing of MOFs.

## Figures and Tables

**Scheme I.**
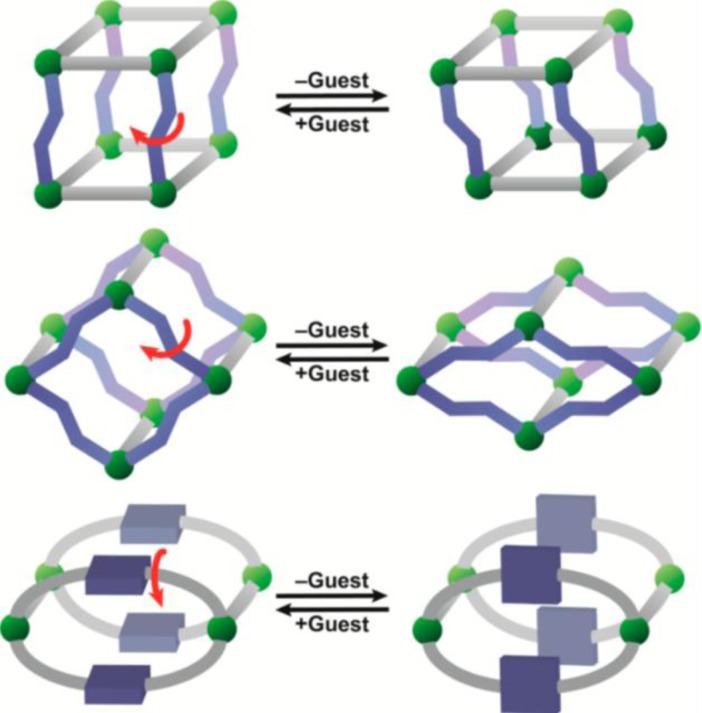
The illustration of “breathing” MOFs synthesized using semi-rigid ligands. Green spheres mean metal nodes, gray rods mean rigid organic ligands, and blue components mean flexible or rotating ligands. Reprinted with permission from [166]. Copyright 2014 American Chemical Society.

**Table 1.** MOFs Synthesized from Hydrothermal Conditions.

MOF Class	Metal Center	Organic Linker	Mineralizing Agent	Temperature (°C)	Time (h)	Pore Diameter (Å)	Space Group	Bravis Lattice	Reference
MIL-47	VCl_3_	BDC	–	200	96	*as*: 7.9 × 12.0 *ht*: 10.5 × 11.0	*as: Pnma* *ht: Pnma*	*as*: orthorhombic *ht*: orthorhombic	[47]
MIL-53	Al(NO_3_)_3_·9H_2_O	BDC	–	220	72	*as*: 7.3 × 7.7 *ht*: 8.5 × 8.5 *lt*: 2.6 × 13.6	*as: Pnma* *ht: Imma* *lt: Cc*	*as*: orthorhombic *ht*: orthorhombic *lt*: monoclinic	[26]
MIL-69		NDC	KOH	210	16	H: 2.7 × 19.4 DeH: 8.5 × 8.5	*C*2/c	Monoclinic	[48]
MIL-96		BTC	–	210	24	Cavity free: 8.8 Pore opening: 2.5–3.5	*P*6_3_/*mmc*	Hexagonal	[49]
MIL-100	Fe metal		HF	160	8	Cage: 25. 29 Window: 5, 9	*Fdm*	Cubic	[50,51]
			HNO_3_		12	–	–	–	[45]
	FeCl_3_ 6H_2_O		–						
	Fe(NO_3_)_3_ 9H_2_O								
	Al(NO_3_)_3_·9H_2_O		HNO_3_	210	3.5	5.2 × 8.8	*Fd*3*m*	Cubic	[52]
	Cr metal		HF	220	96	Internal: 6.6; External: 25			[50]
MIL-110	Al(NO_3_)_3_·9H_2_O		HNO_3_	210	72	16	*P*6_3_22	Hexagonal	[53]
MIL-118	Al(NO_3_)_3_·9H_2_O	H_4_BTEC	–	210	24	*as*: 7.3 × 7.7 *ht*: 8.5 × 8.5 *lt*: 2.6 × 13.6	*as: C*2*/c* *ht: Pbam* *lt: Pnam*	*as*: monoclinic *ht*: orthorhombic *lt*: rthorhombic	[54]
MIL-120	Al(NO_3_)_3_·9H_2_O	H_4_BTEC	NaOH	210	24	5.4 × 4.7	*C*2*/c*	monoclinic	[55]
MIL-121	Al(NO_3_)_3_·9H_2_O	H_4_BTEC	–	210	24	8.7 × 5.7	*C*2*/c*	monoclinic	[40]
MIL-122	Al(NO_3_)_3_·9H_2_O	H_4_NTC	–	210	24	–	*P*2_1_/*c*	monoclinic	[56]
	Ga(NO_3_)_3_·*x*H_2_O								
^*^	In(NO_3_)_3_·5H_2_O								
	CuSO_4_·5H_2_O	L	KBr	85	1.5	Hydrated: 18.99 × 15.14 Dehydrated: 18.56 × 12.81	*P*	triclinic	[11]
Cu-MOF-SiF_6_	CuSiF_6_	BPED	–	rt	72	13.5 × 13.5	*P*4/*ncc*	Tetragonal	[57]
ELM-12	Cu(OTf)_2_	4,4′-bpy	MeOH	rt	>168	11 × 11.5	*Pbna*	Orthorhombic	[58]
			EtOH				*Pn*2_1_*a*		
			1-PrOH				*Pnna*		
			1-BuOH						
^*^	Cu(NO_3_)_3_·3H_2_O	HDHBC 4,4′-bpy	Diethyl ether	rt	a few days	3.6 × 4.2	*P*2/*c*	Monoclinic	[59]

4,4′-bpy = 4,4′-bipyridine; BDC = 1,4-benzene-dicarboxylic acid; BTC = 1,3,5-benzene-tricarboxylic acid; BPED = *meso*-1,2-bis(4-pyridyl)-1,2-ethanediol; H_4_BTEC = 1,2,4,5-benzene-tetracarboxylic acid; H_4_NTC = 1,4,5,8,-naphthalene-tetracarboxylic acid; HDHBC = 2,5-dihydroxybenzoic acid; L = 4,4′-(1,4-(trans-2-butene)diyl)bis(1,2,4-triazole); NDC = 2,6-naphthalene-dicarboxylic acid; OTf = trifluoromethanesulfonate; rt: room temperature

* haven’t been named.

**Table 2.** MOFs Synthesized from Solvothermal Conditions.

MOF Class	Metal Center	Organic Linker	Solvent	Mineralizing Agent	Temprature (°C)	Time (h)	Pore Diameter (Å)	Space Group	Bravis Lattice	Reference
MOF-5	Zn(NO_3_)_2_·6H_2_O	BDC	DMF	C_6_H_15_N; C_6_H_5_Cl; H_2_O_2_	rt	0.5–4	8	*Fm*3*m*	Cubic	[60,61]
	Zn(OAc)_2_·2H_2_O	BDC	DMF	C_6_H_15_N	rt	2.5	7.56	*Fm*3*m*	Cubic	[62]
MOF-74	Zn(OAc)_2_·2H_2_O	H_4_DHTP	DMF	–	rt	18	–	–	–	[62]
	Zn(NO_3_)_2_·4H_2_O			2-Propanol H_2_O	105	20	10.3 × 5.5	$R\bar{3}$	Trigonal	[63]
MOF-177	Zn(NO_3_)_2_·6H_2_O	H_3_BTB	DEF	–	100	23	10.8	$P\bar{3}1c$	Trigonal	[62,64]
MOF-199 (HKUST-1)	Cu(NO_3_)_2_·2.5H_2_O	BTC	DMF	C_6_H_15_N; EtOH; H_2_O	85	24	Cage: 13.2 × 11.1; Aperture: 6.9	*Fm*3*m*	Cubic	[64–66]
IR-MOF-0	Zn(OAC)_2_·2H_2_O	ADC	DMF	C_6_H_15_N	rt	over-night	–	*Fm*3*m*	Cubic	[62]
MIL-88	FeCl_3_·6H_2_O	BDC	DMF	–	150	0.2	3 × 14	$P\bar{6}2c$	Hexagonal	[67,68]
^*^	Co(CF_3_SO_3_)_2_	H_2_BDP	DEF	–	150	144	–	*C*2*/c*	Monoclinic	[69]
CFA2	Cu(OAc)_2_·H_2_O	H_2_PHBPZ	DEF; MeOH	–	110	72	12–14.5	*I*4_1_/*a*	Tetragonal	[70]
CFA3	Ag_2_O	H_2_PHBPZ	EtOH	NH_4_OH	rt	120	–	*P*2_1_/*c*	Monoclinic	[70]
^*^	Zn(NO_3_)_2_·6H_2_O	TCPOM 4,4′-bpy	DMF	–	100	24	–	*C*2*/c*	Monoclinic	[71]
^*^	Zn(NO_3_)_2_·6H_2_O	BDC DABCO	DMF	–	120	48	7.5 × 7.5	*I*4/*mcm*	Tetragonal	[72,73]
^*^	Cd(NO_3_)_2_·4H_2_O	BPNDC 4,4′-bpy	DMF	–	120	24	Cavity: 12 × 7 × 4; Aperture: 2 × 6	*C*2/*c*	Monoclinic	[74]
^*^	La(NO_3_)_2_·6H_2_O	H_3_BTB	DMF	–	95	48	–	*P*6_5_22	Hexagonal	[75]
^*^	Zn(NO_3_)_2_·6H_2_O	2,5-BME-BDC; DABCO	DMF	–	120	48	–	*C*2/*m*	Monoclinic	[76]
^*^	Zn(NO_3_)_2_·6H_2_O	2,3-BME-BDC; DABCO	DMF	–	120	48	–	*C*2/*m*	Monoclinic	[76]
^*^	Zn(NO_3_)_2_·6H_2_O	DE-BDC; DABCO	DMF	–	120	48	–	*C*2/*m*	Monoclinic	[76]
^*^	Zn(NO_3_)_2_·6H_2_O	DP-BDC; DABCO	DMF	–	120	48	–	*C*2/*m*	Monoclinic	[76]
^*^	Zn(NO_3_)_2_·6H_2_O	DiP-BDC; DABCO	DMF	–	120	48	–	*C*2/*m*	Monoclinic	[76]
^*^	Zn(NO_3_)_2_·6H_2_O	DB-BDC; DABCO	DMF	–	120	48	–	*P*4/*mmm*	Tetragonal	[76]
^*^	Zn(NO_3_)_2_·6H_2_O	DPe-BDC; DABCO	DMF	–	120	48	–	*I*4/*mcm*	Tetragonal	[76]
^*^	Zn(NO_3_)_2_·6H_2_O	BA-BDC; DABCO	DMF	–	120	48	–	*P*4/*mmm*	Tetragonal	[76]
^*^	Zn(NO_3_)_2_·6H_2_O	BPy-BDC; DABCO	DMF	–	120	48	–	*P*4/*mmm*	Tetragonal	[76]
CID-23	Zn(NO_3_)_2_·6H_2_O	BPA; IP	DMF	–	120	48	5.6 × 8.6	$P\bar{1}$	triclinic	[77]
FMOF-1	AgNO_3_	NaTz	MeOH	–	rt	n/a	12.2 × 7.3; 4.9 × 6.6	$I\bar{4}2d$	Tetragonal	[78,79]
^*^	Zn(OAc)_2_·2H_2_O	H_3_PBC; 2,2’-bpy	DMF	–	170	96	4.1 × 4.1; 2.7 × 2.7	*C*2/*c*	Monoclinic	[80]
UCY-3	Cd(NO_3_)_2_ 4H_2_O	H_3_CIP	DMF	–	100	20	5–7	*C*2/*c*	Monoclinic	[81]
FMOF-2	Zn(NO_3_)_2_·6H_2_O	H_2_hfipbb	DMF; EtOH	–	110	a few hours	–	*P*2/*n*	Monoclinic	[82]
SUMOF-6	Ln(NO_3_)_6_ salt	H_2_BPYDC	DMF	–	120	24	19.0 × 5.3	*P*2/*n*	Monoclinic	[83]
^*^	CuCl_2_·2H_2_O	H_2_DMCAPZ	DMF; MeOH	–	90	24	6.7 × 7.2(br)9.7 × 9.6	*I*4_1_*md*	Tetragonal	[84]
^*^	Zn(NO_3_)_2_·6H_2_O	BDC; BTZ; PYZ	DMA; MeOH	–	120	120	13.5 × 8.5	*C*2*m*	Monoclinic	[85]
^*^	Zn(NO_3_)_2_·6H_2_O	BDC; TZ; PYZ	DMF	–	120	120	14.5 × 5.0	$P\bar{1}$	Triclinic	[85]

2,2’-bpy = 2,2’-bipyridine; 2,5-BME-BDC = 2,5-bis(2-methoxyethoxy)-1,4-benzenedicarboxylic acid; 2,3-BME-BDC = 2,3-bis(2-methoxyethoxy)-1,4-benzenedicarboxylic acid; 4,4′-bpy = 4,4′-bipyridine; ADC = Acetylenedicarboxylic acid; BA-BDC = 2,5-Bis(allyloxy)-1,4-benzenedicarboxylic acid; BDC = 1,4-benzene-dicarboxylic acid; BPA = 1,4-bis(4-pyridyl)acetylene; BPNDC = Benzophenone-4,4′-dicarboxylate; BPy-BDC = 2,5-Bis(prop-2-ynyloxy)-1,4-benzenedicarboxylic acid; BTC = 1,3,5-benzene-tricarboxylic acid; BTZ = 1H-benzotriazole; DABCO = 1,4-diazabicyclo-[2.2.2]octane; DB-BDC = 2,5-di-butoxy-1,4-benzenedicarboxylic acid; DE-BDC = 2,5-di-ethoxy-1,4-benzenedicarboxylic acid; DEF = *N*,*N*-diethylformamide; DP-BDC = 2,5-di-propoxy-1,4-benzenedicarboxylic acid; DPe-BDC = 2,5-di-pentoxy-1,4-benzenedicarboxylic acid; DiP-BDC = 2,5-di-iso-propoxy-1,4-benzenedicarboxylic acid; DMA = *N*,*N*-dimethylacetamide; DMF = *N*,*N*-dimethylformamide; EtOH = Ethanol; H_2_BDP = 1,4-benzene-dipyrozolate; H_2_BPYDC = 2,2’-bipyridine-5,5’-dicarboxylic acid; H_2_DMCAPZ = 3,5-Dimethyl-4-Carboxypyrazole; H_2_hfipbb = 2,2’-bis(4-carboxyphenyl)hexafluoropropane; H_2_PHBPZ = 3,3’,5,5’-tetraphenyl-1*H*,1’*H*-4,4′-bipyrazole; H_3_CIP = 5-(4-carboxybenzylideneamino)isophthalic acid; H3PBC = 4-phosphono-benzoic acid; H_3_BTB = Benzenetribenoic acid or 1,3,5-tris(4-carboxyphenyl)benzene acid; H_4_DHTP = 2,5-dihydroxyterephtalic acid; IP = Isophthalic acid; MeOH = Methanol; NaTz = sodium perfluorinated ligand 3,5-bis(trifluoromethyl)-1,2,4-triazolate; PYZ = pyrazine; TCPOM = Tetrakis[4-(carboxyphenyl)oxamethyl]methane; TZ = 1,2,3-1H-triazole

* haven’t been named.

**Table 3.** Summary Physical Properties of “Breathing” MIL-53s.

MOF class	Absorbent inside channels	Channel Diameter (Å)	Space group	Unit cell dimension (Å)			Bravis Lattice	Surface Area (m^2^·g^−1^)	Unit Cell Volume (Å^3^)	Reference
				*a*	*b*	*c*				
Al-MIL-53 *as*	Disordered templating BDC	7.3 × 7.7	*Pnma*	17.129	6.628	12.182	Orthorhombic	–	1383.1	[26,111,112]
Al-MIL-53 *ht*	Empty	8.5 × 8.5	*Imma*	6.608	16.675	12.813	Orthorhombic	–	1411.9	[26,112,113]
Al-MIL-53 *lt*	H_2_O	2.6 × 13.6	*Cc*	19.513	7.612	6.576	Monoclinic	1592 (Langmuir) 1140 (BET)	946.8	[26,111,112]
Al-MIL-53	CO_2_	–	–	6.59	18.14	10.38	–	–	1234.2	[113]
Fe-MIL-53 *ht*	Empty	6.759	*C*2/*c*	21.269	6.759	6.884	Monoclinic	–	899.6	[114]
Fe-MIL-53 *lt*	H_2_O	7.518	*C*2/*c*	19.320	15.036	6.835	Monoclinic	–	1973.5	[106,115]
Cr-MIL-53 *as*	Disordered templating BDC	8	*Pnam*	17.340	12.178	6.822	Orthorhombic	1400 (Langmuir)	1440.6	[27,116,117]
Cr-MIL-53 *ht*	Empty	8	*Imcm*	16.733	13.038	6.812	Orthorhombic	–	1486.1	[27,116,117]
Cr-MIL-53 *lt*	H_2_O	–	*C*2/*c*	19.685	7.849	6.782	Monoclinic	––	1012.0	[27,116,117]

Cr-MIL-53 LP	CO_2_	–	*C*2/*c*	19.713	8.310	6.806	Monoclinic	–	1072.0	[27,116,117]
	C_2_H_5_OH			19.621	9.301	6.811			1185.1	[118]
	CH_3_OH			19.641	9.151	6.811			1168.1	

Cr-MIL-53 HP	CH_3_OH	–	*Imcm*	16.131	13.961	6.831	Orthorhombic	–	1538.1	[118]
	CO_2_			16.439	13.500				1527.3	[27,116,117]
	C_2_H_5_OH			16.151	13.971				1541.1	[118]

Ga-MIL-53 *ht*	H_2_O	–	*Imma*	6.717	16.678	13.209	Orthorhombic	1462 (Langmuir) 1140 (BET)	1479.7	[119]

Ga-MIL-53 *lt*	H_2_O	–	*C*2*c*	19.833	6.856	6.714	Monoclinic	–	886.3	[119]

Sc-MIL-53-*cp* (100 K)	Empty	–	*P*2_1_/*c*	20.298	7.331	11.691	Monoclinic	–	1680.8	[120]

Sc-MIL-53-*cp* (573 K)	Empty	–	*P*2_1_/*c*	20.538	7.299	12.560	Monoclinic	–	1804.9	[120]

Sc-MIL-53-*vnp* (623 K)	Empty	–	*C*2*c*	21.505	6.630	7.274	Monoclinic	–	950.83	[120]
